# Real-time learning of predictive recognition categories that chunk sequences of items stored in working memory

**DOI:** 10.3389/fpsyg.2014.01053

**Published:** 2014-10-06

**Authors:** Sohrob Kazerounian, Stephen Grossberg

**Affiliations:** Graduate Program in Cognitive and Neural Systems, Department of Mathematics, Center for Adaptive Systems, Center for Computational Neuroscience and Neural Technology, Boston UniversityBoston, MA, USA

**Keywords:** category learning, working memory, capacity limits, Masking Field, Magical Number 7, speech perception, Adaptive Resonance Theory, Time Invariant String Kernel

## Abstract

How are sequences of events that are temporarily stored in a cognitive working memory unitized, or chunked, through learning? Such sequential learning is needed by the brain in order to enable language, spatial understanding, and motor skills to develop. In particular, how does the brain learn categories, or list chunks, that become selectively tuned to different temporal sequences of items in lists of variable length as they are stored in working memory, and how does this learning process occur in real time? The present article introduces a neural model that simulates learning of such list chunks. In this model, sequences of items are temporarily stored in an Item-and-Order, or competitive queuing, working memory before learning categorizes them using a categorization network, called a Masking Field, which is a self-similar, multiple-scale, recurrent on-center off-surround network that can weigh the evidence for variable-length sequences of items as they are stored in the working memory through time. A Masking Field hereby activates the learned list chunks that represent the most predictive item groupings at any time, while suppressing less predictive chunks. In a network with a given number of input items, all possible ordered sets of these item sequences, up to a fixed length, can be learned with unsupervised or supervised learning. The self-similar multiple-scale properties of Masking Fields interacting with an Item-and-Order working memory provide a natural explanation of George Miller's Magical Number Seven and Nelson Cowan's Magical Number Four. The article explains why linguistic, spatial, and action event sequences may all be stored by Item-and-Order working memories that obey similar design principles, and thus how the current results may apply across modalities. Item-and-Order properties may readily be extended to Item-Order-Rank working memories in which the same item can be stored in multiple list positions, or ranks, as in the list ABADBD. Comparisons with other models, including TRACE, MERGE, and TISK, are made.

## 1. Introduction

### 1.1. Overview: temporary storage of item sequences in working memory and learning of list chunks

Two critical processes in many intelligent behaviors are the temporary storage of sequences of items in a working memory, and the learned unitization of these sequences into recognition categories, also called *list chunks*. The former process uses fast activations of cells and storage of these activities in short-term memory, or STM. The latter process learns to compress, or unitize, the events stored in STM into list chunks, and remembers them using long-term memory, or LTM. These working memory STM and list chunking LTM processes are needed for linguistic, spatial, and motor behaviors. For example, during speech and language, the stored sequence may be derived from pre-processed auditory signals, and the learned list chunks may represent phonemes, syllables, words, and other familiar linguistic units. During motor control, the stored sequence may be motor gestures, and the learned list chunks may represent skilled action sequences. During spatial navigation, the stored sequence may be the locations of desired goal objects on a route, and the learned list chunks may represent plans to carry out the movements to attain a desired goal via this route.

This article develops a model of how sequences of items that are temporarily stored in a working memory are unitized through learning into list chunks. It simulates how learning enables list chunks to become selectively tuned to different temporal sequences of items, and simulates how STM storage and chunk LTM may be carried out in real time in response to lists of variable length. In a network with a given number of input items in a list, model simulations show how all possible ordered sets of these item sequences, up to a fixed length, can be learned with unsupervised or supervised learning.

The present article builds upon established neural models of working memory and list chunking. Previous articles have not shown, however, how list chunk learning can occur in real time as events are stored sequentially in working memory. Providing this crucial missing piece is the current article's main accomplishment.

The article justifies the choice of its particular working memory and list chunk models by reviewing a subset of the psychological and neurobiological data that have been explained and predicted by these models, and the larger cognitive and neural literatures to which the models contribute. The new results about list chunk learning clarify how these models can learn the categorical representations that have been previously used to explain these various data.

**Figure 1:**
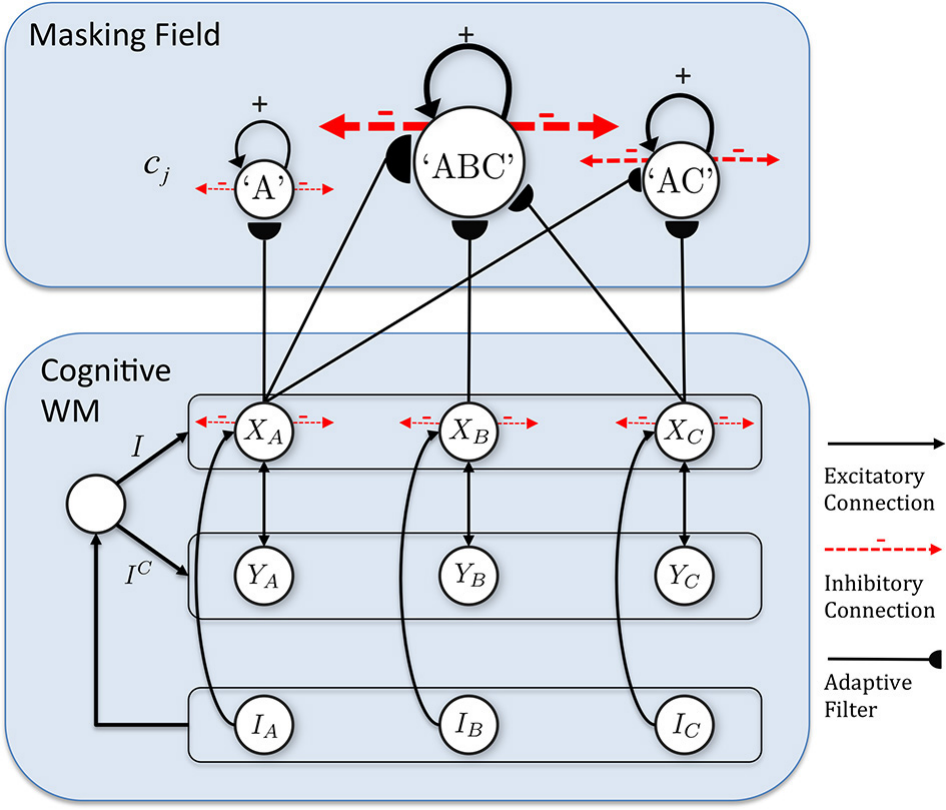
**Macrocircuit of the list chunk learning model simulated in the current article.** An Item-and-Order working memory for the short-term sequential storage of item sequences activates a Masking Field network through an adaptive filter whose weights learn to selectively activate Masking Field nodes in response to different stored item sequences and to thereby convert them into list chunks.

The model that is further developed in the current article describes an Item-and-Order, or competitive queuing, working memory, and a Masking Field list chunking network. The working memory activates list chunks through an adaptive filter whose weights learn to activate different categories in response to different stored sequences of items in the working memory (Figure 1).

Sections 2–4 provide scholarly background about working memory and list chunking data and models. Section 5 describes six new properties that enable learning of list chunks by the model in real time. Section 6 defines the model mathematically. Section 7 describes the computer simulations of list chunk learning. Section 8 describes model extensions, other data explained by the model, and a comparative analysis of other neural models, notably models of speech. Section 9 describes some future directions for additional model development.

## 2. Item-and-order working memory

### 2.1. Primacy gradient in working memory

When we experience sequences of events through time, they may be temporarily stored in a working memory (WM). Tests of immediate serial recall (ISR), in which subjects are presented with a list of items and subsequently asked to reproduce the items in order, were among the early probes of the properties of WM (e.g., Nipher, 1878; Ebbinghaus, 1913; Conrad, 1965; Murdock, 1968; Healy, 1974; Henson, 1996; Wickelgren, 1966).

**Figure 2:**
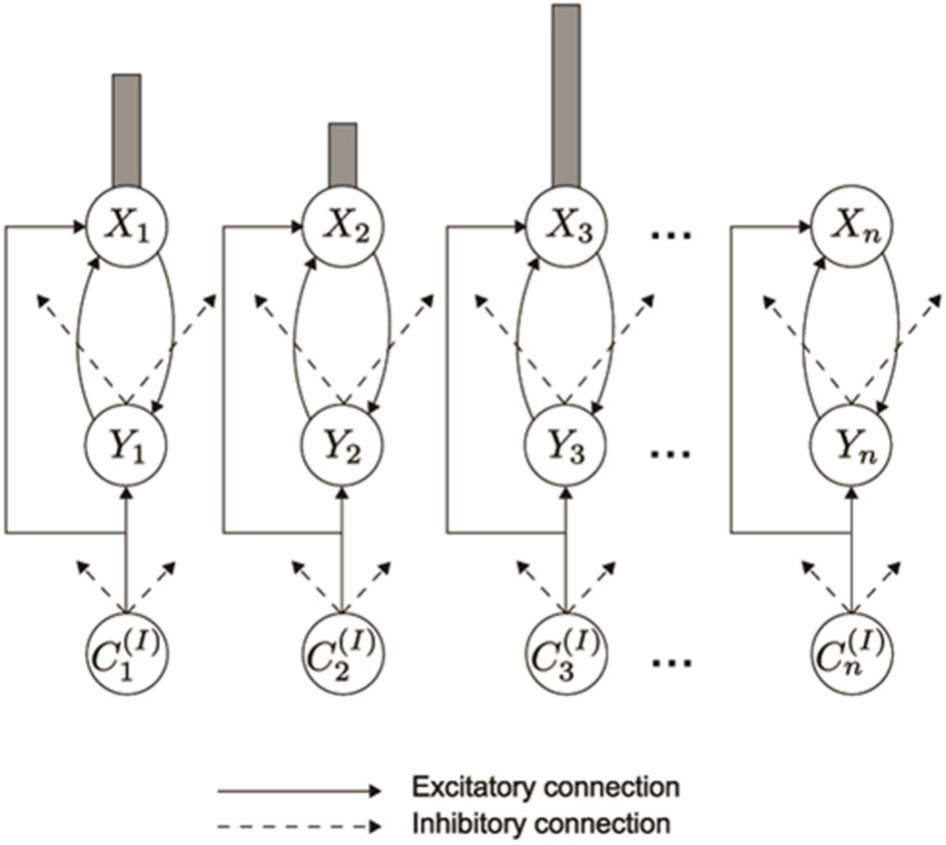
**In an Item-and-Order working memory, acoustic item activities *C*^(*I*)^_1_, *C*^(*I*)^_2_, *C*^(*I*)^_3_, are stored in working memory by a gradient of activity.** A correct temporal order is represented by a primacy gradient, with the most active cell activity *X*_*i*_ corresponding to the first item presented, the second most active corresponding to the second item presented, and so on. (Reprinted with permission from Grossberg and Kazerounian, 2011).

As data accumulated from studies involving ISR and similar tasks, models of WM were developed to explain them. Lashley (1951) suggested that items are retained in parallel in spatially separable neural populations, thus transforming the temporal problem of serial order into a spatial problem. Grossberg (1978a,b) developed a rigorous neural model of WM through which a temporal stream of inputs could be stored as an evolving spatial pattern of item representations (Figure 2), before these patterns are unitized through learning into list chunk representations that can be used to control context-sensitive behaviors. This WM model is called an Item-and-Order model. In it, individual nodes, or cell populations, represent list *items* and the *order* in which the items are presented is stored by an *activity gradient* across the nodes. An item is more properly called an *item chunk*, which, just like any chunk, is a compressed representation of a spatial pattern of activity within a prescribed time interval. In the case of an item chunk, the spatial pattern of activity exists across acoustical feature detectors that process sounds through time. The prescribed time interval is short, and is commensurate with the duration of the shortest perceivable acoustic inputs, of the order of 10–100 msec. Some phonemes may be coded as individual items, but others, in which two or more spatial patterns are needed to identify them, are coded in working memory as a short sequence of item chunks, and are fully unitized as a list chunk. Thus, the model in Figure 1 first compresses spatial patterns of feature detectors into item chunks, and then sequences of these item chunks that are spatially stored in WM are compressed into list chunks.

**Figure 3:**
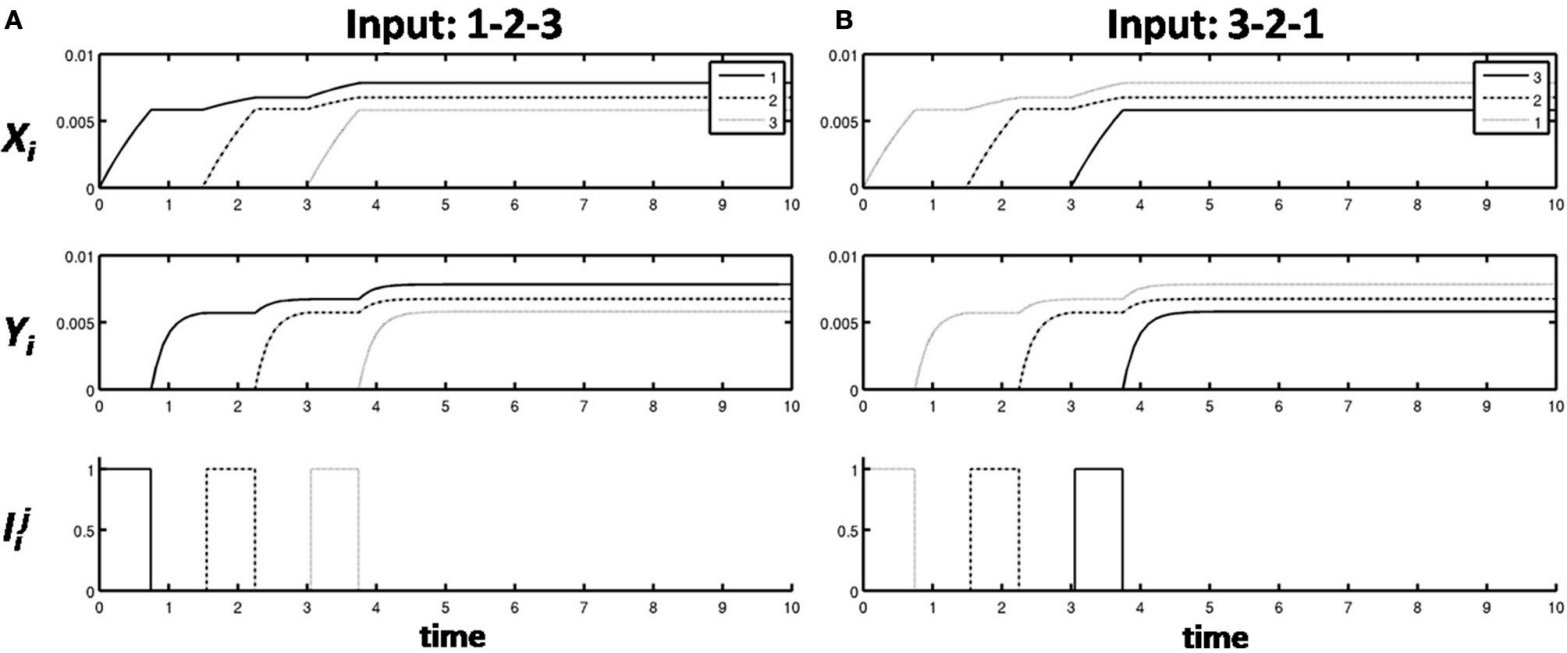
**(A)** A primacy gradient is stored in response to the sequence of items “1-2-3” is shown, with activities in a solid line corresponding to “1,” activities in dashed lines corresponding to “2,” and activities in dotted lines corresponding to “3.” **(B)** A primacy gradient is stored in response to the sequence “3-2-1.”

A *primacy gradient* stores items in WM in the correct temporal order. In a primacy gradient, the first item in the sequence activates the corresponding item chunk with the highest activity, the item chunk representing the second item has the second highest activity, and so on, until all items in the sequence are represented. For example, a sequence “1-2-3” of items is transformed into a primacy gradient of activity with cells encoding “1” having the highest activity, cells encoding “2” with the second highest activity, and cells encoding “3” having the least activity (Figure 3A). Item-and-Order working memories can easily store sequences composed of the same items presented in different orderings. For example, if the sequence “3-2-1” is presented, then “3” has the highest activity, and so on (Figure 3B). Phonemes, syllables, and words can all be coded as sequences of item chunks, before they are unitized into list chunks at the next level of processing.

### 2.2. Rehearsal and inhibition of return

How is a stored spatial pattern in WM used to recall a sequence of items performed through time? A rehearsal wave that is delivered uniformly, or non-specifically, to the entire WM enables read-out of stored activities. The node with the highest activity is read out fastest and self-inhibits its WM representation. By inhibiting the item that is currently being read out, such self-inhibition realizes the cognitive concept of *inhibition of return*, which prevents perseveration on the earliest item to be performed. This self-inhibition process is repeated until the entire sequence is reproduced in its correct order and there are no active nodes left in the WM.

### 2.3. Competitive queuing and primacy models

Since the Grossberg (1978a,b) introduction of this type of model, many modelers have used it and variations thereof (e.g., Houghton, 1990; Boardman and Bullock, 1991; Bradski et al., 1994; Page and Norris, 1998; Bullock and Rhodes, 2003; Grossberg and Pearson, 2008; Bohland et al., 2010). In particular, the Item-and-Order WM is also known as the Competitive Queuing model (Houghton, 1990). Page and Norris (1998) presented a Primacy model to explain and simulate cognitive data about immediate serial order working memory, notably experimental properties of word and list length, phonological similarity, and forward and backward recall effects.

### 2.4. Data about item-and-order storage and recall

Both psychophysical and neurophysiological data have supported the Grossberg (1978a,b) predictions that neural ensembles represent list items, encode the order of the items with their relative activity levels, and are reset by self-inhibition. For example, Farrell and Lewandowsky (2004) did psychophysical experiments in humans that study the latency of responses following serial performance errors. They concluded that (p. 115): “Several competing theories of short-term memory can explain serial recall performance at a quantitative level. However, most theories to date have not been applied to the accompanying pattern of response latencies… Data from three experiments show that latency is a negative function of transposition displacement, such that list items that are reported too soon (ahead of their correct serial position) are recalled more slowly than items that are reported too late. We show by simulation that these data rule out three of the four representational mechanisms. The data support the notion that serial order is represented by a primacy gradient that is accompanied by suppression of recalled items.”

**Figure 4:**
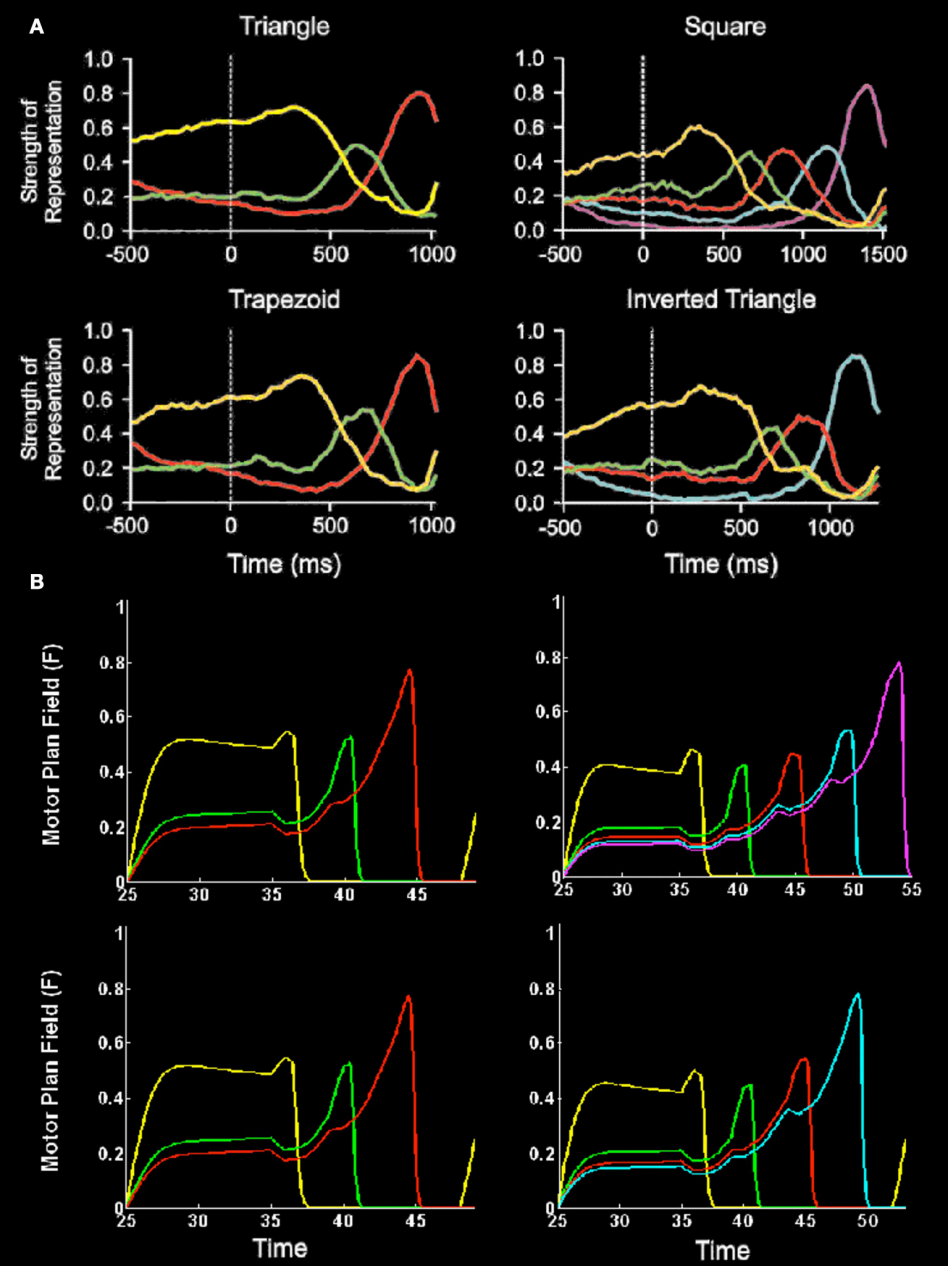
**Neurophysiological data and simulations of monkey sequential copying data. (A)** Plots of relative strength of representation (a complex measure of cell population activity, as defined by Averbeck et al., 2002) vs. time for four different produced geometric shapes. Each plot shows the relative strength of representation of each segment for each time bin (at 25 ms) of the task. Time 0 indicates the onset of the template. Lengths of segments were normalized to permit averaging across trials. Plots show parallel representation of segments before initiation of copying. Further, rank order of strength of representation before copying corresponds to the serial position of the segment in the series. The rank order evolves during the drawing to maintain the serial position code. At least four phases of the Averbeck et al. (2002; **Figure 9A**) curves should be noted: (1) presence of a primacy gradient; that is, greater relative activation corresponds to earlier eventual execution in the sequence during the period prior to the initiation of the movement sequence (period −500–400 ms); (2) contrast enhancement of the primacy gradient to favor the item to be performed (greater proportional representation of the first item) prior to first item performance (period ~100–400 ms); (3) reduction of the chosen item's activity just prior to its performance and preferential relative enhancement of the representation of the next item to be performed such that it becomes the most active item prior to its execution (period ~400 ms to near sequence completion); and (4) possible re-establishment of the gradient just prior to task completion. (Reproduced with permission from Averbeck et al., 2002). **(B)** Simulations of item activity across the motor plan field of the LIST PARSE model for 3, 4, and 5 item sequences vs. simulation time. In both **(A)** and **(B)**, line colors correspond to representations of segments as follows: yellow, segment 1; green, segment 2; red, segment 3; cyan, segment 4; magenta, segment 5. (Reproduced with permission from Grossberg and Pearson, 2008).

Electrophysiological data have also supported these predicted properties, notably from recordings in the peri-principalis region of dorsolateral prefrontal (PFC) cortex in macaque monkeys while they perform action sequences to copy geometrical shapes (e.g., Averbeck et al., 2002, 2003a,b). The predicted properties of a primacy gradient and a self-inhibitory form of inhibition of return were evident in these data. Figure 4 summarizes these data and a simulation of it by the Item-and-Order LIST PARSE model of Grossberg and Pearson (2008).

### 2.5. Bowed gradients during free recall

What is the longest list that the brain can store in working memory in the correct temporal order? In an Item-and-Order working memory, this question translates into: What is the longest primacy gradient that the working memory can store? In particular, can arbitrarily long lists be stored with primacy gradients, and if not, why not? One reason for an upper limit on correct recall is that, as more and more items are stored, the differences in item activations tend to get smaller and smaller, thereby making it harder to differentiate item order, especially if the cells that store the activities are noisy.

There is, in addition, an even more basic reason why only relatively short lists can generate a primacy gradient in working memory, which is reflected in the fact that relatively short lists can be stored with the correct temporal order *in vivo*. Indeed, in free recall tasks, a *bowed* serial position curve is often observed (Ebbinghaus, 1913; Murdock, 1962; Postman and Phillips, 1965; Glanzer and Cunitz, 1966; Tan and Ward, 2000). In these tasks, as a subject repeats a sufficiently long list in any order after hearing it, the items at the beginning and the end of the list are performed earliest, and with the highest probability of recall.

Grossberg (1978a,b) noted that such data have a natural explanation if the WM gradient that stores the list items is also bowed, with the first and last items having the largest activities, and items in the middle having less activity. If the item with the largest activity is read out first, whether at the list beginning or end, and then self-inhibits its item representation to prevent preservation, then the next largest item will be read out, and so on in the order of item relative activity. The greater probability of items being recalled at the beginning and end of the list also has a simple explanation, since items that are stored with larger activities have greater resilience against perturbation by cellular noise.

### 2.6. Magical numbers four and seven: immediate and transient memory spans

What is the longest primacy gradient that can be stored? The classical Magical Number Seven, or *immediate memory span*, of 7 ± 2 items that is found during free recall (Miller, 1956) estimates the upper bound. Grossberg (1978a) argued for a distinction to be made between the immediate memory span and a *transient memory span* that was predicted to be the result of a short-term working memory recall without the benefit of top-down long-term memory read-out. That is, the transient memory span is the longest list for which a primacy gradient may be stored in short-term memory solely as the result of bottom-up inputs. In contrast, the immediate memory span was predicted to scale with the longest primacy gradient that could be stored due to the combined effect of bottom-up inputs and top-down read-out of learned expectations from list chunks (see Section 8). Based on these considerations, the prediction was made, given an estimated immediate memory span of approximately seven items, that the transient memory span should be expected to be approximately four items. Cowan (2001) has since summarized data showing that, when the influences of long-term memory and grouping effects are minimized, there is indeed a working memory capacity limit of 4 ± 1 items. There is thus also a Magical Number Four, as predicted.

## 3. LTM invariance principle: linking working memory STM and list chunk LTM

Why is the transient memory span so short? To explain this, Grossberg (1978a,b) argued that all working memories for the short-term storage of items are designed to enable learning and stable memory of list chunks, and showed that two simple postulates imply these properties: the LTM Invariance Principle and the Normalization rule. Item-and-Order working memories were derived from these postulates.

The LTM Invariance Principle implies that novel sequences of items may be stored and chunked through learning in a way that does not destabilize memories of previously learned chunk subsequences. Without such a property, longer chunks (e.g., for MYSELF) could not be learned without risking the unselective destruction of previously learned memories of shorter chunks (e.g., for MY, SELF, and ELF). Language, motor, and spatial sequential skills would then be impossible. In particular, the LTM Invariance Principle insists that, if bottom-up inputs have activated a familiar subset list chunk, such as the word MY, the arrival of the remaining portion SELF of the novel word MYSELF during the next time interval will not alter the activity pattern of MY in a way that would destabilize the previously learned weights that activate the list chunk of MY. This principle is achieved mathematically by preserving the *relative activities*, or ratios, between working memory activities as new items are presented through time. Newly arriving inputs may, however, alter the *total activities* across the working memory.

The Normalization Rule insists that the total activity of the working memory network be bounded by a maximal finite activity that is independent of the number of items stored in working memory. This normalization property gives rise to the limited capacity of working memory by redistributing, rather than simply adding, activity when new items are stored.

Grossberg (1978a) mathematically proved that, if both the LTM Invariance Principle and the Normalization Rule hold in a working memory, then there is a transient memory span; that is, lists no longer than the transient memory span can be stored as a primacy gradient and thus recalled in their correct temporal order. If a list is longer than the transient memory span, the primacy gradient that is initially stored will evolve into a bowed gradient as more items are stored. In other words, the ability of a working memory to enable learning and stable memory of stored sequences implies an upper bound on the length of lists that can be stored in the correct temporal order.

These results hold when the same amount of attention is paid to each item as it is stored. Indeed, from a purely mathematical perspective, a primacy gradient, recency gradient, or a bowed gradient will be stored, depending on the choice of parameters, where the earliest items to be stored by a recency gradient are the last ones to be performed. If attention is not uniform across items, then multi-modal bows can occur, as during Von Restorff (1933) effects, also called isolation effects (Hunt and Lamb, 2001), which occur when an item in a list “stands out like a sore thumb” and is thus more likely to be remembered than other list items. Associative and competitive mechanisms that are consistent with the Item-and-Order working memory model have been used to explain Von Restorff effects during serial verbal learning (Grossberg, 1969, 1974).

One might worry that postulates such as the LTM Invariance Principle and the Normalization Rule are too sophisticated to be discovered by evolution. These concerns were allayed by the demonstration that both the LTM Invariance Principle and the Normalization Rule can arise within a ubiquitous neural design; namely, a recurrent on-center off-surround network of cells that obey the membrane equations of neurophysiology, otherwise called shunting dynamics. Bradski et al. (1994) proved mathematical theorems about how the length and depth of the primacy, recency, and bowed gradients of such a recurrent on-center off-surround network may be controlled by network parameters.

The fact that linguistic, spatial, and motor sequences, in humans and monkeys, seem to obey the same working memory laws provides accumulating evidence for the Grossberg (1978a,b) prediction that all working memories have a similar design because they all need to obey the LTM Invariance Principle. List chunks in all these modalities can then be learned and stably remembered, and the working memories can be realized by variations of recurrent shunting on-center off-surround networks. See Section 8 for further discussion.

## 4. Masking field

### 4.1. Multiple-scale working memory to chunk variable-length lists

**Figure 5:**
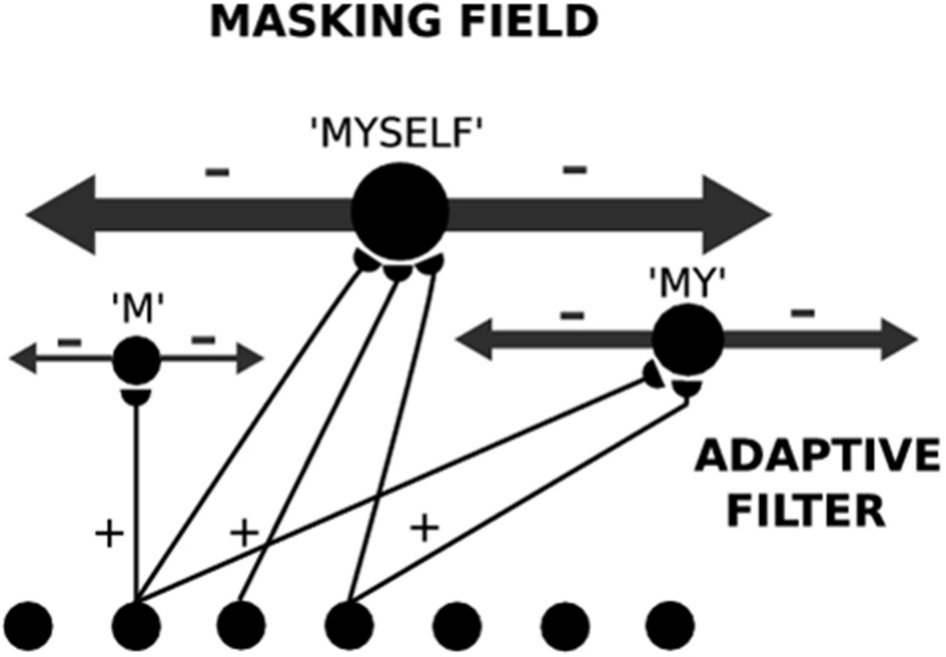
**A Masking Field is shown for three unitized lists which code the sequences “M,” “MY,” and “MYSELF.”** Larger Masking Field cells code longer sequences. Larger cells also have stronger inhibitory connections that enable longer unfamiliar lists to overcome the salience of shorter familiar lists. These asymmetric inhibitory coefficients can arise from self-similar activity-dependent growth laws.

A Masking Field is a specialized type of Item-and-Order working memory. It is also defined by a recurrent on-center off-surround network whose cells obey the membrane equations of neurophysiology. In a Masking Field, however, the “items” are list chunks that are selectively activated by prescribed sequences of item chunks that are stored in an Item-and-Order WM at an earlier processing level (Figure 5). In other words, Masking Field cells are said to represent list chunks because each of them is activated by a particular temporal sequence, or list, of items that is stored within the Item-and-Order working memory at the previous processing level. Thus, both levels of the item and list processing hierarchy are composed of working memories that obey similar laws. In order for Masking Field list chunk to represent lists of multiple lengths, its cells interact within and between multiple spatial scales, with the cells of larger scales capable of selectively representing item sequences of greater length, and of inhibiting other Masking Field cells that represent item sequences of lesser length.

In summary, a network with at least two processing levels is envisaged (Figures 1, 5). In the first level, an Item-and-Order working memory stores sequences of items as they are presented through time. The temporally evolving spatial patterns of stored activity generate output signals through a bottom-up adaptive filter that can learn to activate specific list chunks. This article is devoted to the study of how this adaptive filter learns to selectively activate list chunks as items are stored in working memory through time.

### 4.2. Temporal chunking problem

Masking Fields were introduced to solve the *temporal chunking problem* (Cohen and Grossberg, 1986, 1987; Grossberg, 1978a, 1984) which asks how an internal representation of an unfamiliar list of familiar speech units—for example, a novel word composed of familiar phonemes or syllables—can be learned under the type of unsupervised learning conditions that are the norm during daily experiences with language. Before the novel word, or list, can fully activate the adaptive filter, all of its individual items must first be presented. By the time the entire list is fully presented, all of its sublists will have also been presented. What mechanisms prevent the familiarity of smaller sublists, which have already learned to activate their own list chunks, from forcing the novel longer list to always be processed as a sequence of these smaller familiar chunks, rather than eventually as a newly learned unitized whole? How does a not-yet-established word representation overcome the salience of already well-established phoneme or syllable representations to enable learning of the novel word to occur?

### 4.3. Self-similarity implies asymmetric length-sensitive competition

A Masking Field accomplishes this by assuming that its multiple scales are related to each other by a property of *self-similarity*; that is, each scale's properties, including their excitatory and inhibitory interaction strengths, are a multiple of the corresponding properties in another scale. In particular, larger list chunks represent longer lists and have stronger interaction strengths. The intuitive idea is that, other things being equal, the longest lists are better predictors of subsequent events than are shorter sublists, because the longer list embodies a more unique temporal context. As a result, the *a priori* advantage of longer, but unfamiliar, lists enables them to compete effectively for activation with shorter, but familiar, sublists, thereby suggesting a solution of the temporal chunking problem.

### 4.4. Self-similar laws for activity-dependent development

The same type of question about evolution arises for Masking Fields as arose for Item-and-Order working memories. How can such asymmetric masking coefficients develop in a way that does not require too much intelligence on the part of the evolutionary process? In fact, the asymmetric masking coefficients between Masking Field cells, as well as other self-similar properties, can develop as a result of simple activity-dependent growth laws (Cohen and Grossberg, 1986, 1987). During such a developmental process, some Masking Field cells start out, due to random growth of connections, with more connections from working memory items than do others. It is assumed that there is a developmental critical period during which cells in the item working memory are endogenously active. Masking Field cells that happen to receive more bottom-up connections from these active cells also receive larger total inputs, on average. It is assumed that this activity energizes growth of the recipient list chunk cells and their output connections. This growth is self-similar in two senses: First, the activated list chunk cells grow until they attain the same threshold density of average input through time. As a result, Masking Field cells that receive larger average inputs—that is, respond to longer lists—will grow larger than cells that receive smaller inputs—that is, respond to shorter lists. Second, all parts of a cell grow proportionally, so that as a cell grows larger, its inhibitory connections to other cells in the Masking Field grow stronger too.

As a result of self-similar development, a larger cell can inhibit cells that code subsequences of the items that activate it, more than conversely. This property is realized by asymmetric inhibitory coefficients [see Section 6, Equation (6)], in which the normalized inhibitory effect of the *k*th list chunk *c*_*k*_ on the *j*th list chunk *c*_*j*_ scales with the number of items |*K*| which contact *c*_*k*_ through the bottom-up adaptive filter, and the number of items |*K* ∩ *J*| which input to both chunks.

Masking Field list chunk cells that survive this asymmetric competition best represent the sequence of item chunks that is currently stored in working memory. These asymmetric coefficients thus enable the Masking Field to encode stored item sequences into the biggest list chunks that it can represent. In this sense, the Masking Field encodes the best “prediction” of what item sequence is stored in working memory.

### 4.5. Reconciling response selectivity and predictivity

In addition to an asymmetric competitive advantage for larger cells, and thus longer lists, over smaller cells, and thus shorter lists, each Masking Field cell responds *selectively* to a sequence of a prescribed length. This property ensures that a cell that is tuned to a sequence of length n cannot become strongly active in response to a subsequence of its inputs whose length is significantly less than n. Were this to happen, then the biggest chunks would always win, even if there was insufficient evidence for them. Instead, each cell accumulates evidence from its inputs until enough evidence has been received for the cell to fire. To accomplish this, the total input strength to a Masking Field chunk is normalized by the property of *conserved synaptic sites* (Cohen and Grossberg, 1986, 1987), which is also a consequence of the self-similar growth of cells: Cells that receive more inputs grow larger and thereby dilute the effects of each input until a critical threshold is reached at which all inputs need to fire to activate the cell. In this way, Masking Fields reconcile the potentially conflicting demands of selectivity and predictivity.

## 5. Model heuristics

### 5.1. Six new properties of masking fields

The definition and simulations of a Masking Field in the present article embody a combination of six properties that previous implementations have not incorporated. The simulated Masking Field:

1. Responds to inputs as they occur in *real time*. The Masking Field inputs in the current simulations are not just equilibrium values from an Item-and-Order working memory, as in previous simulations. Instead, the sequences of inputs activate the working memory cells, whose temporally evolving activities, in turn, input to the Masking Field cells in real time.
2. *Contrast normalizes* inputs from the Item-and-Order working memory to the Masking Field using a feedforward shunting on-center off-surround network (Grossberg, 1973, 1978a; Grossberg and Mingolla, 1985; Cohen and Grossberg, 1986; Grossberg and Todorovic, 1988; Heeger, 1992; Douglas et al., 1995). Contrast normalization enables the network to learn the ratios of bottom-up inputs [Grossberg, 1978a, 1980); see Section 6, Equation (6)].
3. Includes *habituative transmitter gates* that exist in the bottom-up pathways of the adaptive filter from the Item-and-Order working memory to the Masking Field [see Section 6, Equations (6) and (7)] These gates are activity-dependent and weaken previously active signals to prevent perseverative activation of the same list chunk for an unduly long time. They thereby facilitate timely reset of list chunks in response to dynamically changing inputs.
  Habituative transmitter gates were introduced into the neural modeling literature in Grossberg (1968) and used to explain data about development, reinforcement learning, and cognition (e.g., Grossberg, 1972, 1976b, 1978a, 1980; Olson and Grossberg, 1998; Grossberg and Seitz, 2003; Dranias et al., 2008; Fazl et al., 2009), visual perception (e.g., Francis et al., 1994; Francis and Grossberg, 1996; Grossberg and Swaminathan, 2004; Grossberg and Yazdanbakhsh, 2005; Berzhanskaya et al., 2007; Grossberg et al., 2008), spatial navigation (Grossberg and Pilly, 2012, 2013; Mhatre et al., 2012; Pilly and Grossberg, 2013), and speech and language (e.g., Grossberg et al., 1997; Grossberg and Myers, 2000). Habituative gates are sometimes called *depressing synapses* after the rederivation of this law from neurophysiological data recorded from visual cortex (Abbott et al., 1997), data which confirmed predicted habituative gate properties.
4. Learns list chunks from these dynamic inputs. In particular, the *competitive instar* learning law in the adaptive filter that connects the Item-and-Order working memory to the Masking Field enables on-line learning whereby chunk cells become selectively tuned to particular sequences in real time.
5. Can learn list chunks which represent *all possible sequences* that can be derived from the inputs that it processes, up to a fixed length. That is, if there are n item chunks represented in the working memory, and a maximal list chunk coding length is 4, then $1!\left(\begin{array}{c} n\\ 1 \end{array}\right)+2!\left(\begin{array}{c} n\\ 2 \end{array}\right)+3!\left(\begin{array}{c} n\\ 3 \end{array}\right)+4!\left(\begin{array}{c} n\\ 4 \end{array}\right)$ list chunks can be learned, where there are 1!$\left(\begin{array}{c} n\\ 1 \end{array}\right)$ sequences of length one, 2!$\left(\begin{array}{c} n\\ 2 \end{array}\right)$ sequences of length two, 3!$\left(\begin{array}{c} n\\ 3 \end{array}\right)$ sequences of length three, 4!$\left(\begin{array}{c} n\\ 4 \end{array}\right)$ sequences of length four, and $\left(\begin{array}{c} n\\ k \end{array}\right)=\frac{n!}{k!(n-k)!}$.
6. Obeys the *LTM Invariance Principle*, and thereby guarantees stable memories of previously learned LTM codes for familiar sublists. In particular, the Item-and-Order working memory that was used in the current simulations is the STORE 2 working memory (Bradski et al., 1994) which responds in real time to an incoming sequence of inputs, and realizes the LTM Invariance Principle and Normalization Rule.

### 5.2. Solving three problems about chunking variable-length primacy gradients

These refinements overcome the following three kinds of problems:

1. Primacy chunking problem. This problem concerns how a Masking Field learns selective list chunks in response to sequences of item inputs of increasing length. Suppose that list items are stored through time in a primacy gradient in the Item-and-Order working memory. As more inputs are presented, items nearer to the end of the sequence are stored by progressively smaller activity levels than items presented earlier in the sequence. While this property facilitates recall of the list in the correct temporal order, it makes it harder to achieve selective choice of Masking Field list chunks in response to long item sequences. This is because, when a small final item activity is passed through the bottom-up adaptive filter, it will have a smaller effect on list chunk activation than earlier item inputs. Some compensatory mechanism is needed to ensure, for example, that a three item input sequence such as “1-2-3,” when augmented with a fourth item “4,” can supplant a Masking Field chunk that selectively responds to 1-2-3 (a 3-chunk) with a chunk that selectively responds to 1-2-3-4 (a 4-chunk), despite the relatively small activity stored by item “4.”
  This problem is rendered more acute when the inputs arrive, and learning occurs, in real time. Because the competitive dynamics of the Masking Field make it a winner-take-all network, the final input “4” must arrive sufficiently quickly, and with enough strength, that the bottom-up inputs to a 4-chunk enable the activity of that chunk to overtake the activity of the currently most active 3-chunk. If the inputs to the Masking Field were static, no such problem would arise, since all list chunk cells would receive the full extent of the input sequence simultaneously, thereby ensuring that no chunk has a momentary temporal advantage over any other. Solving this problem through parameter setting alone is difficult because the required list chunk selectivity must hold for sequences and list chunks of all sizes.
  Habituative transmitter gates in the bottom-up pathways from the working memory to the Masking Field provide a compensatory mechanism that works well when the items are presented sequentially in time. As noted above, habituative gates prevent perseverative activation in feedback networks, and thereby reset them in response to temporally changing inputs. Because early items in working memory are stored for longer periods of time, their bottom-up gates have a longer time to habituate, thereby enabling newly arriving inputs to have a stronger effect on new list chunk selection.
2. Self-similar activation problem. This problem concerns how to ensure that the self-similar design of the Masking Field works effectively in response to lists of variable length. Suppose that a Masking Field contains list chunks that code up to a maximal list length of four items. Then the total number of potential input sequences, and thus list chunks, which could be learned is $1!\left(\begin{array}{c} 4\\ 1 \end{array}\right)+2!\left(\begin{array}{c} 4\\ 2 \end{array}\right)+3!\left(\begin{array}{c} 4\\ 3 \end{array}\right)+4!\left(\begin{array}{c} 4\\ 4 \end{array}\right)=64$. However, if a Masking Field contains list chunks that code up to a maximal list length of eight items, then the total number of list chunks jumps to 2080. To preserve self-similar properties across Masking Fields which code lists of different maximal length, and thus different total input size, inputs from the working memory need to compensate for the variable total number of inputs to their target list chunks. Using a contrast-normalizing feedforward on-center off-surround network to deliver inputs from the item chunks to the list chunks does this compensation, and furthermore facilitates processing of the ratios that help to ensure the LTM Invariance Principle.
3. Noisy filter problem. This problem concerns how to select the values of random noise that are initially added to the bottom-up filter to set the stage for subsequent weight learning, as occurs in all adaptive filters that use competitive learning, self-organizing maps, or Adaptive Resonance Theory (e.g., Carpenter and Grossberg, 1987; Cohen and Grossberg, 1987). In particular, if the mean and variance of the random noise are inappropriately added to the deterministically chosen initial values of weights, then the Masking Field can be too strongly biased toward selecting the same incorrect list chunk in response to arbitrary sequences, despite the action of habituative gates. To illustrate, imagine two Masking Field cells that are connected to the stored items “1,” “2,” “3,” and “4.” Suppose, moreover, that the Masking Field cells receive bottom-up weights from these items that are all initialized to a value of 1, with some small amount of noise added. If the sequence “1-2-3-4” is presented, and the first list chunk wins the competition, the weights to this list chunk will begin to track, and become parallel to, the primacy gradient of activity across the working memory cells. If the next sequence presented is “1-2-4-3,” then the first chunk is again more likely to win the competition due to the fact that newly learned weights arriving from items “1” and “2” are now larger than their initial uniform values, giving it a competitive advantage.

The goal of the current simulations is to show how a Masking Field can learn all possible orderings of item representations, chosen from five items, up to a list length of four. This goal demonstrates how to achieve maximum flexibility, but it is more demanding than many real-world learning scenarios, where only subsets of all possible lists need ever to be learned. For example, if we consider the problem of learning syllables, the rules of English phonology restrict the phonemes which are allowed to occur in the onset, nucleus and coda of a syllable, as in the phoneme /ŋ/ in bang, which cannot occur at the onset of a syllable. Furthermore, allophonic distinctions are made for a phoneme depending on its position, such as /t/ which is aspirated as [*t*^h^] at the beginning of a stressed syllable, but unaspirated after /s/. In the case of *unsupervised* learning, one simple way to enable all lists to be learnable is to construct a single vector of initial random values for all the list chunks of a particular size, and then permute these values to define the initial weights of each cell of the same size. Doing so ensures that no list chunk cells are given an unfair advantage.

A second method is to implement a kind of *supervised* learning that occurs in Adaptive Resonance Theory, or ART. Here, when a predictive mismatch occurs, incorrect list chunk selections can be reset, allowing a search cycle to select the next best candidate list chunk. Both of these methods have been successfully implemented and are shown in the results.

Yet another method, which has not been fully implemented due to excessive simulation times on the order of weeks or more, is to choose a large enough number of Masking Field cells, relative to the number of sequences to be learned, so that the learned list chunks are sparse with respect to the number of potential list chunk cells that can represent each sequence. One of the first mathematical proofs of the utility of sparseness was given in the article that introduced the modern form of the competitive learning model (Grossberg, 1976a). This proof showed that, even without the top-down matching, attention, and search processes of an ART model, sparse inputs could be stably learned. However, even for Masking Field networks with a relatively small number of item representations (e.g., 8 items), this can quickly become a computationally unwieldy problem.

### 5.3. Simulation protocol

To simulate the learning process, each of the ordered sets of items up to length four were enumerated, and presented to the network one sequence per trial. Each trial began with a sequence of items presented at a fixed rate, and was allowed to run for five simulation time steps after a list chunk had been selected. Selection is determined to occur once a list chunk cell activity has reached a firing threshold which ensures that it will win the competition in the Masking Field and thus inhibit all other cells. In the case of supervised learning, selection requires that the cell not only reaches firing threshold, but additionally that it does not get reset due to an error. In both supervised and unsupervised learning simulations, the threshold activity is set to 0.2.

Although simulation results showed that both randomized as well as repeated presentations of sequences were learnable, the results herein used repeated presentation. Learning in the model used a *self-normalizing* instar learning law [Equation (12) (Grossberg, 1976a; Carpenter and Grossberg, 1987)]. Because instar learning drives bottom-up weights toward a stable equilibrium whose value can be directly computed, the amount of learning that has taken place can be determined by looking at the degree of adaptive sharpening that the bottom-up weights undergo across trials.

## 6. Model equations

Readers who wish to skip the mathematical definition of the model can directly read the results in Section 7. The model is defined mathematically as a system of differential equations that describe the fast short-term memory, or STM, activities of Item-and-Order working memory and Masking Field cells; the intermediate medium-term memory, or MTM, habituative gating process that modulates the strength of bottom-up filter inputs to the Masking Field; and the slow long-term memory, or LTM, learning process within the bottom-up adaptive filter from working memory to Masking Field.

### 6.1. STORE working memory

The model Item-and-Order working memory is a STORE 2 network (Bradski et al., 1994), which realizes both the LTM Invariance Principle and the Normalization Rule.

#### 6.1.1. Input sequences

The input *I*_*i*_ to the *i*th item chunk is a unit pulse of duration α. The maximal length of an input sequence is four. If item *I*_*i*_ is in the *j*th position of a sequence, then it is denoted by *I*^*j*^_*i*_, and obeys:

(1) $$I_{i}^{j}=\{\begin{array}{c} 1,(j-1)(\alpha +\beta )<t<j\alpha +(j-1)\beta \\ 0,\text{ otherwise} \end{array}.$$

By (1), each input in a sequence has duration α and inter-stimulus interval β. In all simulations, α = β = 0.75. Thus, if the input sequence is “1-2-3-4,” then the corresponding inputs are “*I*^1^_1_ − *I*^2^_2_ − *I*^3^_3_ − *I*^4^_4_,” whereas if the input pattern is “4-3-2-1,” then the inputs are “*I*^1^_4_ − *I*^2^_3_ − *I*^3^_2_ − *I*^4^_1_.” The responses of STORE 2 activities to input sequences are invariant under large variations in input parameters; see Bradski et al. (1994) for details.

#### 6.1.2. Layer 1 activities

The activity *x*_*i*_ of the *i*th cell in the first layer of the STORE 2 model obeys:

(2) $$\frac{dx_{i}}{dt}=(0.01I_{i}+y_{i}-x_{i}x-0.7x_{i})I,$$

Equation (1) contains an excitatory bottom-up input 0.1*I*_*i*_, a positive feedback signal *y*_*i*_ from the corresponding *i*th cell of the second layer, a non-specific inhibitory off-surround signal *x* = ∑_*k*_*x*_*k*_ that is shunted by the current activity *x*_*i*_ in the inhibitory term −*x*_*i*_*x*, and a passive decay term −0.7*x*_*i*_. The rate of change is gated on and off by the total input *I* = ∑_*k*_*I*_*k*_ to the working memory at any time, so that activities *x*_*i*_ are able to integrate their inputs only when some input *I*_*i*_ is on.

#### 6.1.3. Layer 2 activities

The activity *y*_*i*_ of the *i*th cell in the second layer obeys:

(3) $$\frac{dy_{i}}{dt}=5(x_{i}-y_{i})I^{C},$$

where *I*^*C*^ = 1 − *I*. This equation forces activities *y*_*i*_ to track the activities of *x*_*i*_ only during intervals when *I*^*C*^ = 1 (i.e., all inputs *I*_*i*_ are off, such that *I* = 0).

#### 6.1.4. LTM Invariance in a STORE model

To see why the STORE 2 model achieves LTM Invariance, suppose that item *I*^*j*^_*i*_ is presented after *k* other inputs have already occurred. Because *I*^*j*^_*i*_ = 1, *I* = 1 and *I*^*C*^ = 0, $\frac{dy_{i}}{dt}$ = 0 and:

(4) $$\frac{dx_{i}}{dt}=\left(0.01I_{i}^{j}-x_{i}x-0.7x_{i}\right).$$

During this interval, *x*_*i*_ approaches the value $\frac{0.01}{x+0.7}$. For the other *k* items (*k* < *i*) already presented, assuming that the integration rates are quick with respect to the inter/intra-input intervals, *y*_*k*_ ≅ *x*_*k*_(*t* − β); that is, when the *j*th item in the sequence is being presented, all *y*_*k*_ are approximately equal to the value *x*_*k*_ had reached at the end of the *previous* item presentation. Because of this, by (2):

(5) $$\frac{dx_{k}}{dt}≅x_{k}(t-\beta )-x_{k}x-0.7x_{k}.$$

By (5), each of the activities *x*_*k*_ approaches $\frac{x_{k}\left(t-\beta \right)}{x+0.7}$, so that the working memory activity values of all previously presented items have the same denominator. LTM invariance is hereby demonstrated, because if a stored pattern is perturbed by some newly arriving inputs, the ratio between all previously stored inputs remains fixed.

The strength of the gradient across working memory activity values, as well as whether or not they form a primacy, recency, or bowed gradient, is controlled by the relative strengths of bottom-up input, recurrent feedback, off-surround inhibition, and the decay rate. For a detailed analysis, see Bradski et al. (1994).

### 6.2. Masking field

#### 6.2.1. Masking Field activities

The activity of a list chunk cell *c*_*j*_ that codes the sequence *J* is defined by the shunting recurrent on-center off-surround network:

(6) $$\begin{array}{l} 4\frac{dc_{j}}{dt}=-Ac_{j}+(1-c_{j})\left[R_{j}\left(B\sum_{i\in J}X_{i}Z_{i}W_{ij}+D|J|f{\left(c\right)}_{j}\right)\right]\\ \;-E(c_{j}+F)\left[\text{​}L\sum_{k\neq i}x_{k}Z_{k}W_{kj}+H\frac{\sum_{K}g(c_{k})|K|(1+|K\cap J|)}{\sum_{K}|K|(1+|K\cap |J)}\text{​}\right]\text{​}. \end{array}$$

Equation (6) contains a passive decay term −*Ac*_*j*_, where A = 0.5. The total excitatory input *R*_*j*_(*B*∑_*i* ∈ *J*_ *x*_*i*_*Z*_*i*_*w*_*ij*_ + *D*|*J*|*f*(*c*_*j*_)) is shunted by (1 − *c*_*j*_), which ensures that activity remains bounded above by 1. The value *R*_*j*_ = 1 for all *c*_*j*_, in all unsupervised learning cases. This value changes through time during the reset events of supervised learning, which is discussed below. The excitatory inputs, from left to right, include bottom-up inputs *B* ∑_*i* ∈ *J*_ *x*_*i*_*Z*_*i*_*W*_*ij*_ from the *i*th working memory cell activities *x*_*i*_, which are multiplied by a habituative gate, *Z*_*i*_, and a bottom-up adaptive weight, or long-term memory trace, *W*_*ij*_, which allows its list chunk to be selectively activated due to learning. The parameter *B* = 3.

The off-surround input $L\sum_{k\neq i}x_{k}Z_{k}W_{kj}+H\frac{\sum_{k}g(c_{k})|K|(1+|K\cap J|)}{\sum_{k}|K|(1+|K\cap J|)}$ tends to normalize Masking Field activities, and is shunted by the term *E*(*c*_*j*_ + *F*), which ensures that the activity of the cell remains bounded below by −*F*.

#### 6.2.2. Habituative gates

The rate of change of the habituative gate *Z*_*i*_ in the pathways from working memory cell activity *x*_*i*_ to any list chunk activity *c*_*j*_ is defined by:

(7) $$\frac{dZ_{i}}{dt}=\varepsilon (1-Z_{i})-Z_{i}(\lambda x_{i}+\mu x_{i}^{2}).$$

(Grossberg, 1972; Gaudiano and Grossberg, 1991; Grossberg and Myers, 2000). Function *Z*_*i*_ helps to prevent perseveration of list chunk activations, as discussed above. Term (1 − *Z*_*i*_) says that gating strength passively recovers to its maximum value 1 at rate ε. Term −*Z*_*i*_(λ*x*_*i*_ + μ*x*^2^_*i*_) says that the gate habituates at an activity-dependent rate determined by the strength of the signal *x*_*i*_ and the parameters λ and μ, which specify linear and quadratic rates of activity-dependent habituation. These linear and quadratic terms allow the gated signal *B*∑_*i*∈*J*_ *x*_*i*_*Z*_*i*_*W*_*ij*_ emitted from the cell to exhibit a non-monotonic response, such that, as signal *x*_*i*_ in (7) increases, the gated signal increases as well, until, at high enough *x*_*i*_ levels, it decreases. With only a linear term, the gated signal at equilibrium would be a monotonically increasing function of the input activity *x*_*i*_. The quadratic term facilitates activity-dependent reset of persistently active cells. The parameters for all habituative gating equations were set to ε = 0.01, λ = 0.1, and μ = 3.

#### 6.2.3. Initial weight

Each weight *W*_*ij*_ is initially set equal to:

(8) $$\left[\frac{1}{\left|J\right|}(1-p_{\left|J\right|})+r_{ij}p_{\left|J\right|}\right]$$

(Cohen and Grossberg, 1986, 1987). The deterministic bottom-up contribution $\frac{1}{\left|J\right|}$(1 − *p*_|*J*|_) to the initial weight is normalized by a the scaling factor of $\frac{1}{\left|J\right|}$, which is inversely proportional to the number of inputs |*J*| converging on list chunk, *c*_*j*_, from the sequence *J* that inputs to that chunk from the working memory. The scaling of bottom-up inputs to list chunk cell size by $\frac{1}{\left|J\right|}$ normalizes the maximum total bottom-up input to the cell and hereby realizes conservation of synaptic sites. This property helps Masking Field cells to selectively respond to sequences of different length. It accomplishes this property by preventing cells which code for lists of given length from becoming too active in response to shorter sequences.

The fluctuation coefficient *p*_|*J*|_ in (8) controls the degree of fluctuation in the growth of weights in the bottom-up filter. When *p*_|*J*|_ = 0(1), growth is deterministic (random). The random values *r*_*ij*_ are uniformly distributed between 0 and 1 such that ∑_*j* ∈ *J*_ *r*_*ij*_ = 1. The fluctuation coefficient *p*_|*J*|_ is selected in such a way as to keep the statistical variability of the connection strengths independent of |*J*|. This is done by ensuring that the coefficient of variation (standard deviation divided by the mean) of $\left[\frac{1}{\left|J\right|}(1-p_{\left|J\right|})+r_{ij}p_{\left|J\right|}\right]$ is independent of |*J*| by setting $p_{\left|J\right|}=p\sqrt{\frac{J+1}{J-1}}$ (Cohen and Grossberg, 1986, 1987). For these simulations, $p=\frac{3}{10\sqrt{3}}$.

In the case of unsupervised learning, an additional step balances noise by choosing random noise values which are based on the size of a list chunk, and permuting these values to define the weights arriving at each list chunk of that size. That is, construct a 1 × |*J*| noise vector $\vec{r_{\left|J\right|}}$ and normalize it such that $\sum \vec{r_{\left|J\right|}}=1$. Then, for each of the |*J*|! list chunks with connections from items *J*, the values of *r*_*ij*_ in Equation (8) are set to one of the |*J*|! permutations of $\vec{r_{\left|J\right|}}$. In a Masking Field whose list chunks receive input connections from five items and a maximum sequence length of four, this process results in a vector of random values for sets of length one through four.

#### 6.2.4. On-center feedback

The recurrent on-center term, *D*|*J*|*f*(*c*_*j*_) in (6), may arise due to activity-dependent self-similar growth of cells during a prior developmental period, as described in Section 4.4. This self-excitatory feedback term is proportional to the number |*J*| of cortical inputs received by the list chunk, and helps a Masking Field to achieve selectivity by providing a competitive advantage to cells that receive inputs from longer lists. The parameter *D* = 30. The self-excitatory feedback *f*(*c*_*j*_) is a sigmoid signal function:

(9) $$f(w)=\frac{w^{2}}{w^{2}+f_{0}^{2}}$$

where the half-maximum value 0.5 of the signal function was chosen to occur at *f*_0_ = 0.75.

The inhibitory inputs to a list chunk *c*_*j*_ are shunted by *E*(*c*_*j*_ + *F*), ensuring that activity remains above -F. The inhibitory input contains a feedforward off-surround input *L*∑_*k* ≠ *i*_ *x*_*k*_*Z*_*k*_*W*_*kj*_, which arrives from all working memory cells. This term does not involve a non-local transport of weights when the feedforward on-center off-surround input network is realized by a laminar cortical network (e.g., Grossberg, 1999; Grossberg and Williamson, 2001) wherein the weights converge on a target cell which, in turn, relays them via an on-center off-surround network to the next processing stage. Teaching signals are, in turn, relayed back to the initial stage from more superficial layers of such a network.

#### 6.2.5. Off-surround feedback

The inhibitory recurrent off-surround signals $H\frac{\sum_{k}g(c_{k})|K|(1+|K\cap J|)}{\sum_{k}\left|K\right|(1+|K\cap J|)}$ embody the inhibitory masking coefficients resulting from self-similar growth laws. Here *J* and *K* denote the sequences that activate *c*_*j*_ and *c*_*k*_ respectively; terms |*J*| and |*K*| denote the numbers of items in these sequences; and term |*K* ∩ *J*| denotes the number of items that the two cells share. In all, the inhibitory input to a cell *c*_*j*_ from a neighboring cell *c*_*k*_, is proportional to the signal *g*(*c*_*k*_), where the sigmoid signal function g is defined by:

(10) $$g(w)=\frac{w^{2}}{w^{2}+g_{0}^{2}}$$

multiplied by the size |*K*| of the *k*th cell, and the number of inputs |*K* ∩ *J*| shared by *c*_*k*_ and *c*_*j*_. The half maximum output signal value in (10) is *g*_0_ = 1. The larger value of the half maximum value *g*_0_ of the inhibitory feedback signal than the value *f*_0_ = 0.75 in (9) of the excitatory feedback signal enables the contrast-enhancement of list chunk activities to begin before inhibition sets in too strongly. These inhibitory coefficients help to realize Masking Field selectivity by allowing larger cells to more strongly inhibit smaller cells, with inhibition proportional to the number of items contacting a given list chunk. Shunting inhibition ∑_*k*_|*K*|(1 + |*K* ∩ *J*|) in the denominator of the inhibitory term defines divisive normalization that results in conservation of synaptic sites, which ensures that the maximum total strength of inhibitory connections to each list chunk is equal to 1.

#### 6.2.6. Mismatch reset during supervised learning

Supervised learning enables the masking field to do away with the careful choice of initial bottom-up weights, and in particular, of noise, that was used in the unsupervised learning simulations to ensure that no list chunk would initially gain too strong a competitive advantage over any other. All possible sequences can be learned, without the need to sparsify the learning problem by adding a much larger number of list chunk cells than the number of sequences to be learned. This constraint can be relaxed in the supervised learning scenario, which immediately resets a list chunk cell in the case of an incorrect selection.

During supervised learning, a reset mechanism is activated whenever a predictive error is made. How such a reset mechanism may be realized dynamically as part of a larger network architecture is explained by Adaptive Resonance Theory (Carpenter and Grossberg, 1987, 1991; Grossberg, 2007, 2012; Grossberg and Versace, 2008). For simplicity, reset is here realized algorithmically: an error occurs when a list chunk that has already been committed by prior learning to a particular sequence is later selected in response to a different sequence. The reset function, *R*_*j*_, in Equation (6) equals:

(11) $$R_{j}=f\left(c_{j},c_{j}^{M}\right)=\{\begin{array}{c} 1,c_{j}>0.2,c_{j}^{M}=\emptyset \text{ or }c_{j}^{M}=J\\ 0,\;c_{j}\geq 0,\;c_{j}^{M}\neq J \end{array}.$$

By (11), if the activity *c*_*j*_ exceeds the threshold value 0.2 (which can trigger self-excitatory feedback that drives the selected cell to its maximum value), and its category is either uncommitted (*c*^*M*^_*j*_ = ∅) or is activated by the correct previously learned sequence *J* (*c*^*M*^_*j*_ = *J*), then the category is not reset (*R*_*j*_ = 1) However, if the category is activated by a different sequence than the one to which it was associated through previous learning (*c*^*M*^_*j*_ ≠ *J*), then the category is reset (*R*_*j*_ = 0). Reset gates off the cell's bottom-up input and self-excitatory feedback, thereby allowing a different list chunk to become active.

With supervised reset implemented, the initial random weights $\left[\frac{1}{\left|J\right|}(1-p_{\left|J\right|})+r_{ij}p_{\left|J\right|}\right]$ in (8) can be chosen much more freely: a unique random value *r*_*ij*_ was constructed for each (*i*, *j*) pair, rather than constructing a permuted vector of weights for each cell size |*J*|. The remaining parameters were selected as in the unsupervised case with $p_{\left|J\right|}=p\sqrt{\frac{J+1}{J-1}},p=\frac{3}{10\sqrt{3}},$ and ∑_*j* ∈ *J*_*r*_*ij*_ = 1.

### 6.3. Competitive instar learning

During both unsupervised and supervised learning, the bottom-up adaptive weight, *W*_*ij*_, in (6) from the *i*th item in working memory to the *j*th list chunk is defined by a competitive instar learning equation that self-normalizes the total learned weight abutting each Masking Field cell (Carpenter and Grossberg, 1987):

(12) $$\frac{dW_{ij}}{dt}=\alpha f(c_{j})\left[(1-W_{ij})x_{i}-W_{ij}\sum_{k\neq i}x_{k}\right].$$

The learning rate in (12) is determined by parameter α, and all learning is gated by the positive self-excitatory feedback signal *f*(*c*_*j*_) in (6). This ensures that only list chunk cells with sufficient activity *c*_*j*_ can learn. For sufficiently active cells, learning drives the weights *W*_*ij*_ to track the pattern of activity across the working memory cells with activities *x*_*i*_. Because of the excitatory term (1 − *W*_*ij*_), each weight *W*_*ij*_ attempts to code a proportion of the total weight, 1. The inhibitory term *W*_*ij*_∑_*k* ≠ *i*_*x*_*k*_, ensures that the weights are competitively distributed among the items that activate *c*_*j*_. This fact can be seen by rewriting (12) in the form:

(13) $$\frac{dW_{ij}}{dt}=\alpha f(c_{j})\left[x_{i}-W_{ij}\sum_{k}x_{k}\right].$$

and noting that *W*_*ij*_ is attracted to a time-average of the ratio $\frac{x_{i}}{\sum_{k}x_{k}}$ of activities during times when the gating signal *f*(*c*_*j*_) is positive.

## 7. Simulation results

### 7.1. Masking field selectivity and weighing of sequential evidence

The Masking Field was tuned so that when sequences of length n are stored in working memory, only list chunk cells that receive exactly n inputs are chosen. As inputs are presented sequentially in time, the list chunk cells will attain different levels of activation at different times during the input presentation, until at least one of the cells exceeds the threshold activity at which self-excitatory feedback drives the cell to become maximally active and quench all other cell activities.

Simulations showing selectivity use the model equations described in Section 6 with initial weights chosen for the unsupervised learning case, but with no learning. Because the weights are permuted across list chunks of the same size, it was sufficient to present the input sequences “1,” “1-2,” “1-2-3,” and “1-2-3-4” to the STORE 2 working memory as input pulses, *I*^*j*^_*i*_, which selectively activate the corresponding working memory cell activities [*x*_*i*_ and *y*_*i*_ in Equations (2) and (3)]. Both the input pulse durations and the inter-stimulus intervals (defined by α and β, respectively) are 0.75 simulation time units. Each trial in these simulations was run until a choice was made in the Masking Field. Selectivity was demonstrated for Masking Fields with four item representations, and therefore 64 masking field list chunks, five items with 205 list chunks, six items with 516 list chunks, seven items with 1099 list chunks, eight items with 2080 list chunks, and nine items with 3609 list chunks, without the need for any parameter changes. That is to say, the masking field responses to the sequences “1,” “1-2,” “1-2-3,” and “1-2-3-4” all resulted in the selection of a list chunk of the correct size. The fact that selectivity was obtained across a network whose size changed by more than a factor of 50, without a change of parameters, demonstrates model robustness.

**Figure 6:**
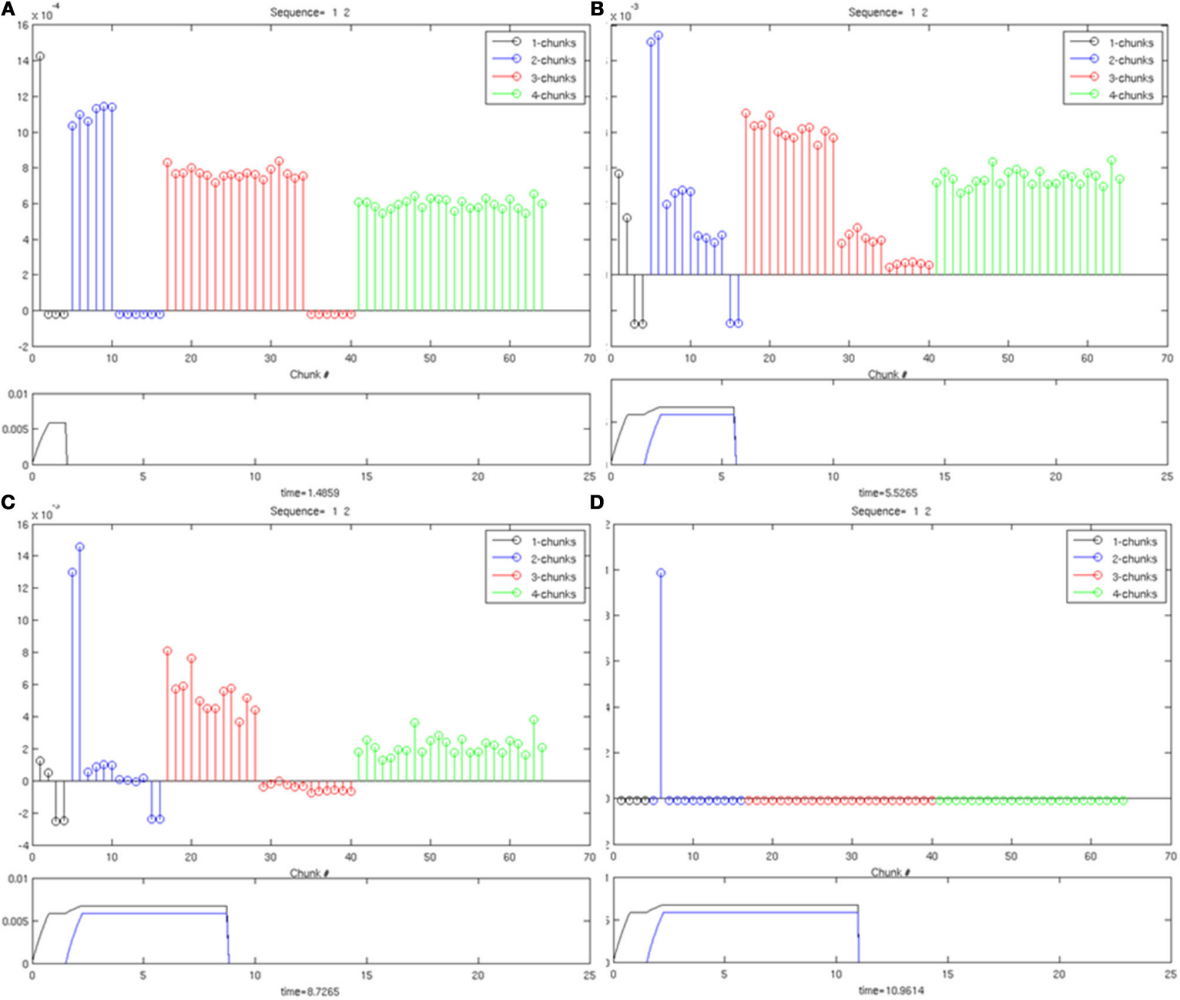
**An example of Masking Field dynamics when two items are stored in working memory.** List chunk activities are shown at various stages of input presentation. In each image, the lower frame shows the inputs to the working memory and the upper frame shows the masking field activities at that time. **(A)** Only one item is presented to working memory. A distributed activity pattern is generated across list chunks representing 1, 2, 3, and 4 items, with the most active cell a 1-chunk. **(B)** When two items are presented to working memory, a 2-chunk is most active. **(C**,**D**): As time continues, without the addition of any new inputs, one of the 2-chunks is selected through winner-take-all dynamics.

The simulation in Figure 6 shows a Masking Field at four points in time, as it is responding to an input sequence of two items being stored in working memory. This masking field only represents four input items, and has 64 list chunk cells. As the first input arrives [activity trace in lower plot of (Figure 6A)], the initial burst of activity in the masking field [stem plot at the top in (Figure 6A)] most strongly activates a 1-chunk, but also activates 2-chunk, 3-chunk, and 4-chunk cells by decreasing amounts, corresponding to the intuition that larger chunks have less evidence to support the hypothesis that they represent. As the second item enters working memory [second activity trace in the lower plot of (Figure 6B)], the 2-chunks begin receiving complete evidence for their list from their bottom-up inputs. Because of the self-similar, asymmetric masking coefficients, the 2-chunk activity overtakes the activity of the 1-chunk plots of (Figures 6B,C) and wins the competition while quenching all other cells [stem plot of (Figure 6D)]. Note the primacy gradients for activation of the first two items at the bottom of (Figures 6A–D).

**Figure 7:**
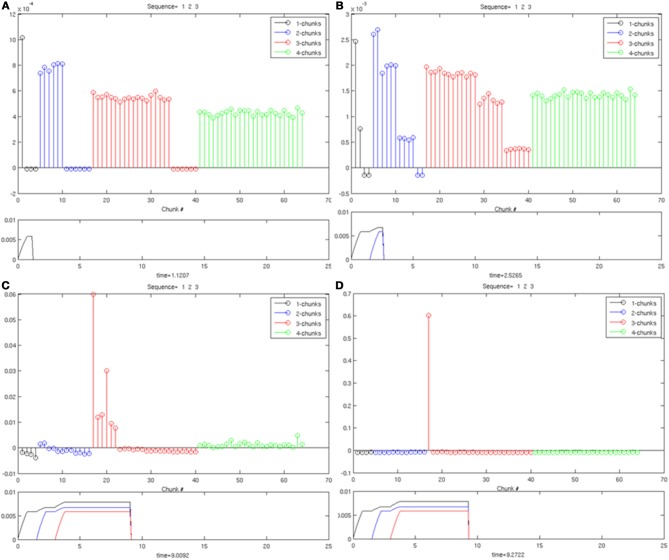
**Same as in Figure 5, with three items stored in working memory. (A)** One item in working memory and a 1-chunk is most active. **(B)** Two items in working memory, and a 2-chunk is most active. **(C)** Three items are stored in working memory, and a 3-chunk is most active. **(D)** As time goes on, a 3-chunk is chosen and all other chunks are inhibited.

**Figure 8:**
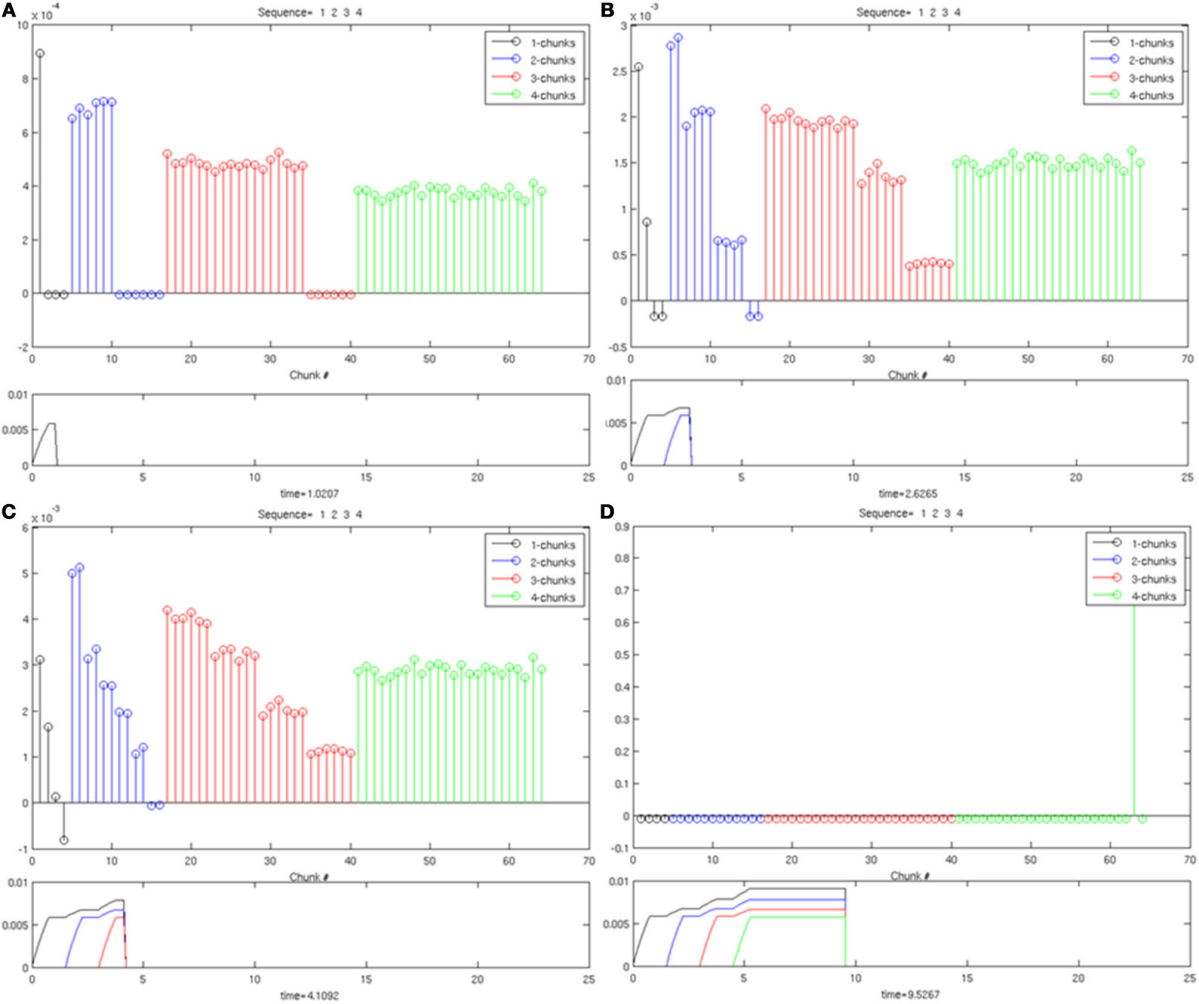
**Same as in Figure 6, with four items stored in working memory. (A)** One item in working memory and a 1-chunk is most active. **(B)** Two items in working memory, and a 2-chunk is most active. **(C)** Three items are stored in working memory, and a 3-chunk is most active. **(D)** shows the winner-take-all choice of a 4-chunk.

In Figure 7, the same Masking Field responds to an input sequence of three items (“1-2-3”). As before, in (Figure 7A), a 1-chunk becomes most active in response to the single item stored in working memory. When the second item begins to be stored in working memory see (Figure 7B), the 2-chunks, now receiving all of their bottom-up activity, start to become the most active list chunk cells. When the third item begins to be stored (Figures 7C,D), the 3-chunks receive all of their bottom-up inputs, and begin to strongly mask their subchunks as a result of their asymmetric inhibitory coefficients. As a result, a list chunk of size three is ultimately selected. The same Masking Field undergoes a similar process with a sequence of four items (“1-2-3-4”) shown in Figure 8, and ultimately a 4-chunk wins the competition across the field. Note the primacy gradients for the activation of the first three items and four items (Figure 8) at the bottom of each figure.

Selectivity obtains, not only when the number of item representations are increased, but also when the number of items remains fixed and additional Masking Field cells are added to create redundant representations. For example, as noted above, for a Masking Field with five item representations, 205 list chunk cells are required to learn all possible orderings of all possible sequences. That is, there are $1!\left(\begin{array}{c} 5\\ 1 \end{array}\right)+2!\left(\begin{array}{c} 5\\ 2 \end{array}\right)+3!\left(\begin{array}{c} 5\\ 3 \end{array}\right)+4!\left(\begin{array}{c} 5\\ 4 \end{array}\right)=205$ different combinations of sequences drawn from five items, up to length 4. More generally, the number of masking field cells required for *m* total items, with *n* redundant copies for each sequence, can be calculated as: $n\left[1!\left(\begin{array}{c} m\\ 1 \end{array}\right)+2!\left(\begin{array}{c} m\\ 2 \end{array}\right)+3!\left(\begin{array}{c} m\\ 3 \end{array}\right)+4!\left(\begin{array}{c} m\\ 4 \end{array}\right)\right]$. In the case of 5 item cells, if there are two potential list chunk cells present for any possible sequence, this yields a total of 410 list chunk cells. Simulations have shown that, in cases of redundant coding, selectivity holds for Masking Fields with four items with one redundant cell (i.e., 64 sequences, with a redundant cell yielding 128 list chunks), four items with two redundant cells (i.e., 64 sequences, with two redundant cells yielding 192 list chunks), five items with one redundant cell (i.e., 205 sequences with one redundant cell yielding 410 list chunks), and five items with two redundant cells (i.e., 205 sequences with two redundant cells yielding 615 list chunks).

Obtaining selectivity without learning sets the stage for a Masking Field to be able to correctly learn sequences. In particular, it is necessary that an input sequence of length |*J*| be capable of selecting a list chunk of the correct size. Lack of selectivity may cause, for example, a list chunk which encodes the sequence “1-2-3” to always become active in response to the input “1-2-3-4.” In such cases, the full sequence cannot be learned correctly. Once selectivity is ensured, a Masking Field can at the very least distinguish between sequences of different lengths. Moreover, once this property is assured, the Masking Field can also select between list chunks of the same length, but which receive different inputs, so long as the weights to the Masking Field are balanced. For example, if one list chunk receives its bottom-up inputs from the items “1,” “2,” “3,” and “4,” and the other from “1,” “2,” “3,” “5,” then when the sequence “1-2-3-4” is presented, the first list chunk will be selected because it receives all of its bottom-up inputs, which will give it a larger total input because of balanced weights. It still must be shown, however, that learning enables presentation of sequences such as “1-2-3-4” and “1-2-4-3” to ultimately choose different masking field cells. This is shown in the next section.

### 7.2. Unsupervised learning

Unsupervised learning simulations demonstrate how all possible sequences that are generated from a fixed number of items can be selectively categorized through learning. In particular, a Masking Field with 205 list chunk cells and five item representations successfully learns to categorize all lists of length one, two, three, or four. The number of items in the network was chosen to be five both to encompass the Transient Memory Span and to avoid too great a combinatorial growth in the number of list chunks as the number of items increases. Such a growth becomes problematic because of the fully connected nature of the masking field. The number of inhibitory signals between cells in a masking field with *n* cells requires *O*(*n*^2^) calculations. For a Masking Field of size four with 64 items, this only represents 4096 calculations. For five items with 205 list chunks, this represents a 10-fold increase, with 42,025 calculations. For eight items, there are 2080 list chunks, and thus the number of inhibitory signals increases more than 1000-fold to 4,326,400 calculations required in a single time step. Because of the increased simulation times of larger networks, and the fact that the previous section showed selectivity from four items (64 list chunks) to 9 items (3609 list chunks) without any parameter changes, our focus in the learning simulations is to confirm that the model choice of bottom-up adaptive filter can support selective learning.

In the selectivity simulations in Section 7.1, the initial weights for the bottom-up filter were chosen in a balanced way to prevent random growth from biasing the competition in favor of particular cells. Such a choice of initial weights also enables unsupervised learning to maintain selectivity while learning optimum weights for all the list chunks. This result was achieved with a small enough learning rate of α = 0.001 [Equation (12)] to avoid a similar imbalance from being created by fast learning. Specifically, just as random noise can bias a competition to favor particular list chunks, so too can a list chunk which has had an opportunity to rapidly learn a sequence such as “1-2-3-4,” just before the presentation of a sequence “1-2-4-3.” If the learning rate is too large, the list chunk 1-2-3-4 can be selected by the sequence “1-2-4-3” over an alternative list chunk whose weights are more uniform. Slow learning was used to prevent any list chunk from getting an undue advantage, and thereby ensuring that every sequence can be selectively learned by a different list chunk without error. In summary, during unsupervised learning, balanced initial weights and slow learning were both imposed to enable the Masking Field to learn all possible sequences up to length four.

We also tested the Masking Field under the weaker condition that not every sequence needed to be learned by distinct list chunks. These simulations were identical to the simulations of unsupervised learning under the strict condition that all sequences be learnable, with the exception of how noise is selected. These simulations again used a Masking Field of five items, with 205 list chunks, slow learning, and all parameters as previously described. In order to add noise to the bottom-up weights, however, a random value *r*_*ij*_ was chosen for each item *x*_*i*_ and list chunk *c*_*j*_, rather than for each list size |*J*|. Weights were then set via the term $\left[\frac{1}{\left|J\right|}(1-p_{\left|J\right|})+r_{ij}p_{\left|J\right|}\right]$. Parameters were set to $p_{\left|J\right|}=p\sqrt{\frac{J+1}{J-1}},p=\frac{3}{10\sqrt{3}},$ and ∑_*j*∈*J*_ *r*_*ij*_ = 1. Simulations showed that between 59 and 66% of the 205 sequences could be correctly learned with these more general initial weights. That is, between 122 and 135 sequences learned to select a unique list chunk. This decrement provides a measure of the improvement that is achieved when supervised learning is used with these more general initial weights (Section 7.3).

To carry out the learning simulations, in both the strict and weak conditions, all 205 sequences of five items were enumerated and presented one per trial. The sequences were repeated cyclically in the same order after every 205 trials. Sequence presentations were constructed by first enumerating the total list of possible sequences, and on the *i*th trial, presenting the sequence denoted by ((i–1) modulo 205) + 1. That is, on the 206th trial, the sequence to be presented was determined by ((206–1) modulo 205) + 1 = 1, such that the 1st sequence in the enumerated list would be used as input. The input sequence was a series of pulses, with the pulse duration and inter-stimulus interval set to 0.75 simulation time units. Each learning trial was ended five simulation time units after a list chunk wins the competition.

**Figure 9:**
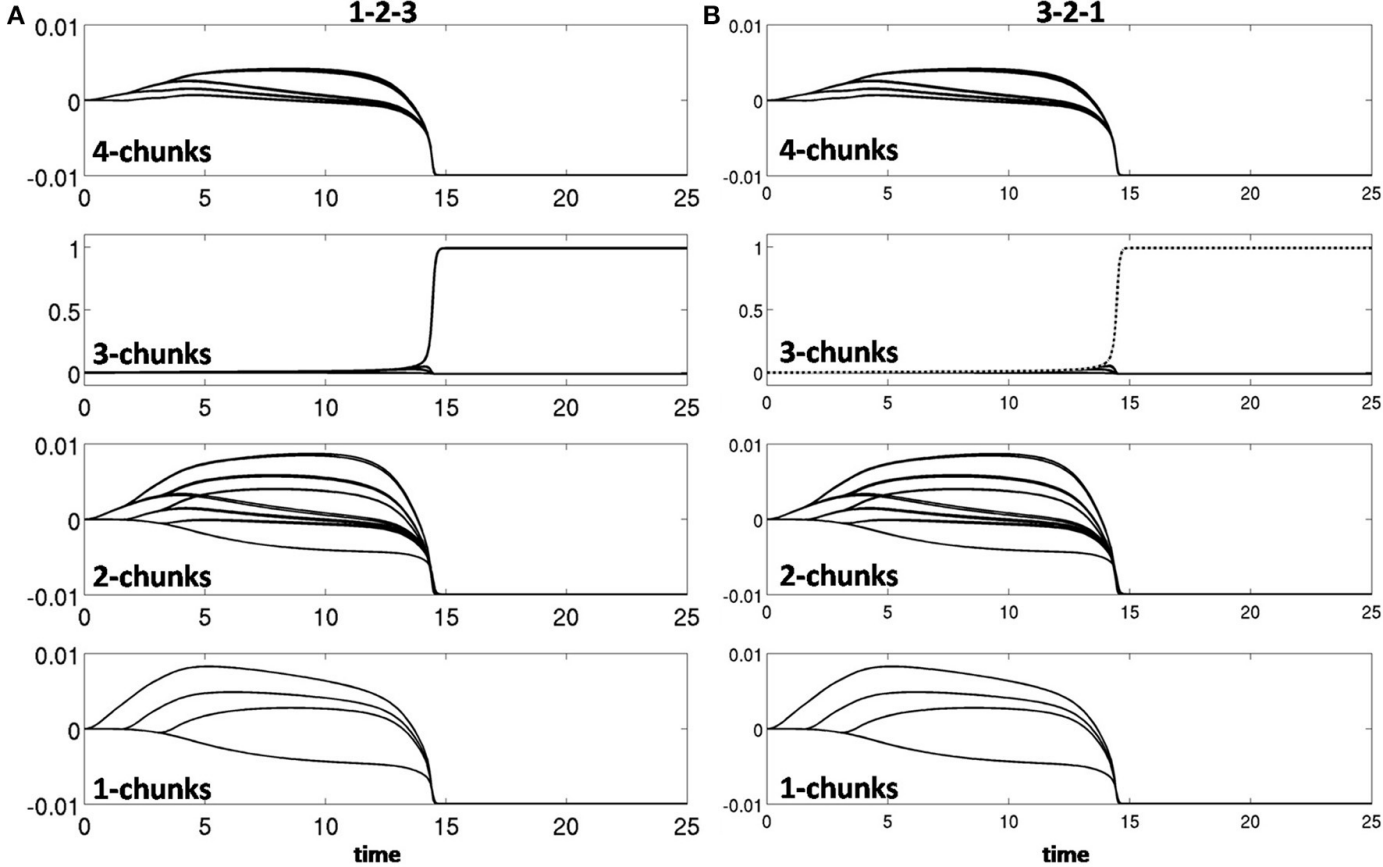
**Activities of the Masking Field through time as it responds to (A) sequence “1-2-3” (left) and (B) sequence “3-2-1.”** In both cases, a 3-chunk is selected (second row). The random noise in the initial bottom-up filter values enable selection of different Masking Field cells in response to sequences of the same items in different orderings.

**Figure 10:**
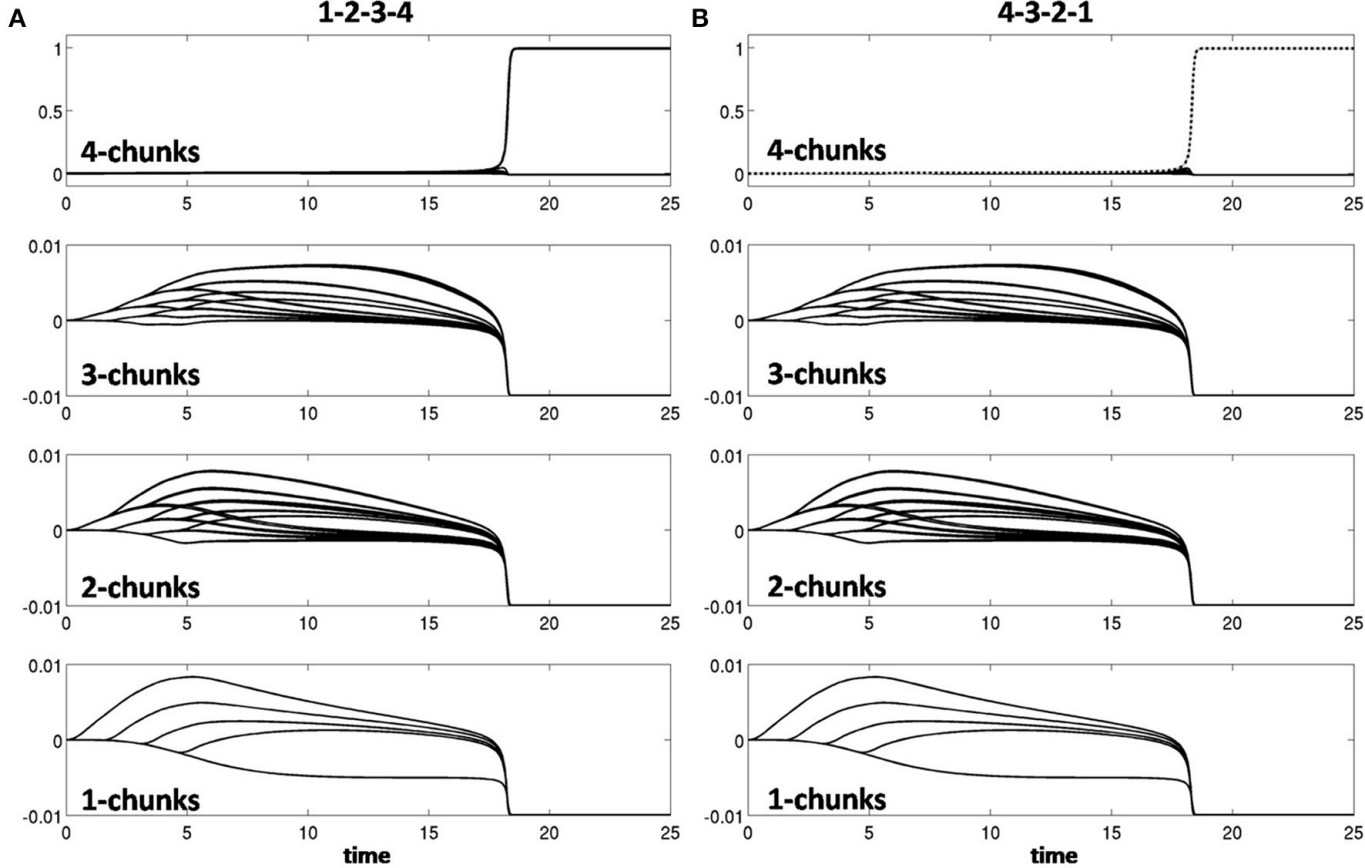
**Winner-take-all chunk choices (first row) by the Masking Field to the sequences (A) “1-2-3-4” and (B) “4-3-2-1.”** Different 4-chunks are chosen to represent the different sequences.

Learning occurred only when an item sequence was presented, and a list chunk was selected by the winner-take-all competition, due to the post-synaptic gating of the instar learning law in (12) by a sigmoid signal function *f*(*c*_*j*_). List chunk dynamics through time before learning occurred are shown for sequences “1-2-3” and “3-2-1” in Figure 9, and for sequences “1-2-3-4” and “4-3-2-1” in Figure 10.

**Figure 11:**
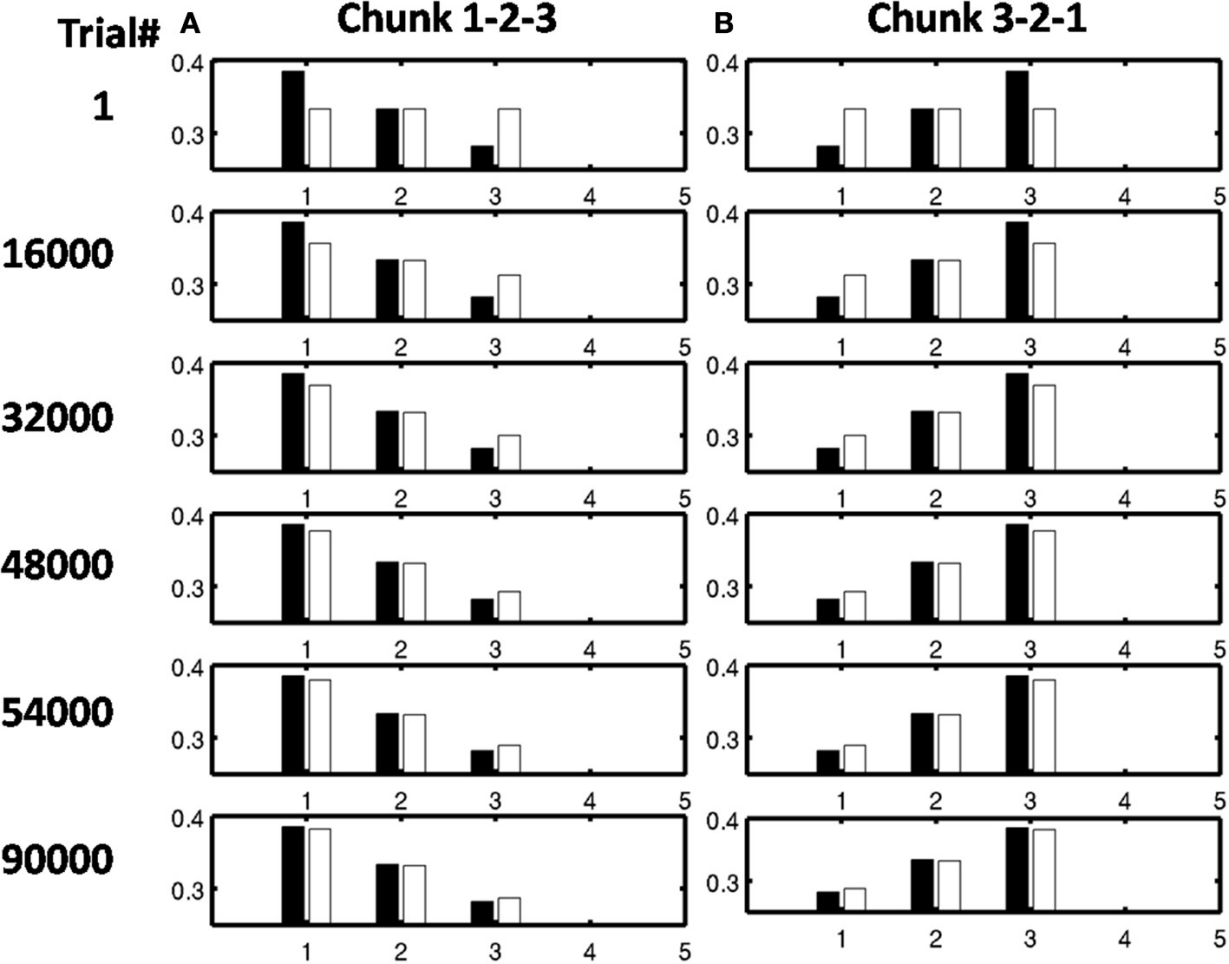
**Adaptive filter weights during learning through time of the list chunks for (A) sequence “1-2-3” and (B) sequence “3-2-1.”** The white bars represent the actual weights to these cells, while the black bars represent the ground truth weights that are expected after learning. At trial 1, the weights to the list chunks are essentially uniform with only the addition of small amounts of noise. Over time, these weights become parallel to the ground truth weights. For these simulations, there are 205 sequence presentations, before any sequence is presented again. By trial 16,000, where a trial is the presentation of a single sequence, each sequence had been presented a total of 77 times; by trial 32,000, 144 times; by trial 48,000, 221 times; by trial 54,000, 298 times; and by trial 90,000, 442 times.

**Figure 12:**
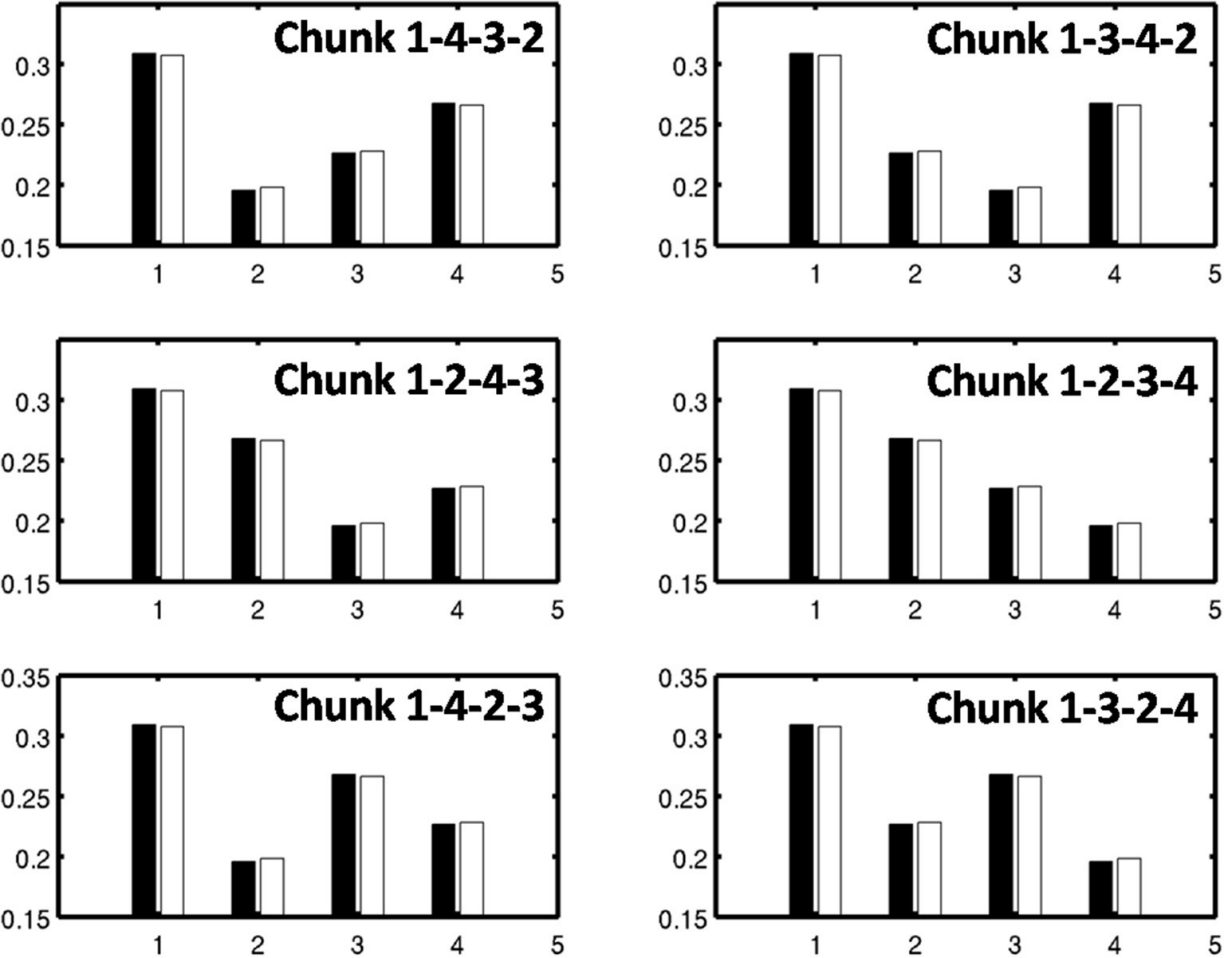
**Learned weights of list chunks that receive inputs from items “1,” “2,” “3,” and “4,” and that represent sequences whose first item is 1.** The learned weights have all converged to the ground truth weights.

As learning proceeds, the weights in the bottom-up adaptive filter to the Masking Field become tuned to the spatial patterns of activity in working memory. For any Masking Field cell that becomes sufficiently activated, the self-normalizing instar law drives that cell's weights to become parallel to the normalized pattern of activity across working memory which activated it. To track the progress of learning, a comparison is made between the normalized pattern of activity across working memory to the learned weight patterns through time. Figure 11 shows the weights for the list chunk cells that code sequences “1-2-3” in column 1 and “3-2-1” in column 2, as they change over the course of the simulation (white bars) to match the corresponding input signals (black bars). Figure 12 shows the final weights for the six chunk cells which receive bottom-up inputs from “1,” “2,” “3,” and “4,” whose first item is “1.”

### 7.3. Supervised learning

As noted in Section 6, during supervised learning, the Masking Field is reset when a list chunk cell that has previously categorized one sequence is subsequently selected in response to a different sequence. Reset enables learning without the need to choose balanced weights. Instead, noise is added to the initial weights by defining $W_{ij}=\left[\frac{1}{\left|J\right|}(1-p_{\left|J\right|})+r_{ij}p_{\left|J\right|}\right]$, where *r*_*ij*_ is a random variable chosen from a uniform distribution, and normalized such that ∑_*j* ∈ *J*_ *r*_*ij*_ = 1. The remaining parameters were chosen as in unsupervised learning ($p_{\left|J\right|}=p\sqrt{\frac{J+1}{J-1}},p=\frac{3}{10\sqrt{3}}$).

The supervised learning simulation also uses a Masking Field with five items and 205 sequences. If a sequence is presented, and a list chunk wins the competition (exceeds the threshold activity of 0.2), if the list has not previously been selected in response to any other sequence, the chunk is allowed to learn for five simulation time units, at which point the trial ends. If, however, the selected list chunk has previously been associated with a *different* sequence, the activity the cell is reset, and the resulting disinhibition of other cells enables another list chunk to win. This reset, or search, process continues until a list chunk is selected which has not previously been committed to an alternate sequence than the one currently being presented. Again, after a list chunk is selected, it is allowed to learn for five simulation time units until the end of the trial.

**Figure 13:**
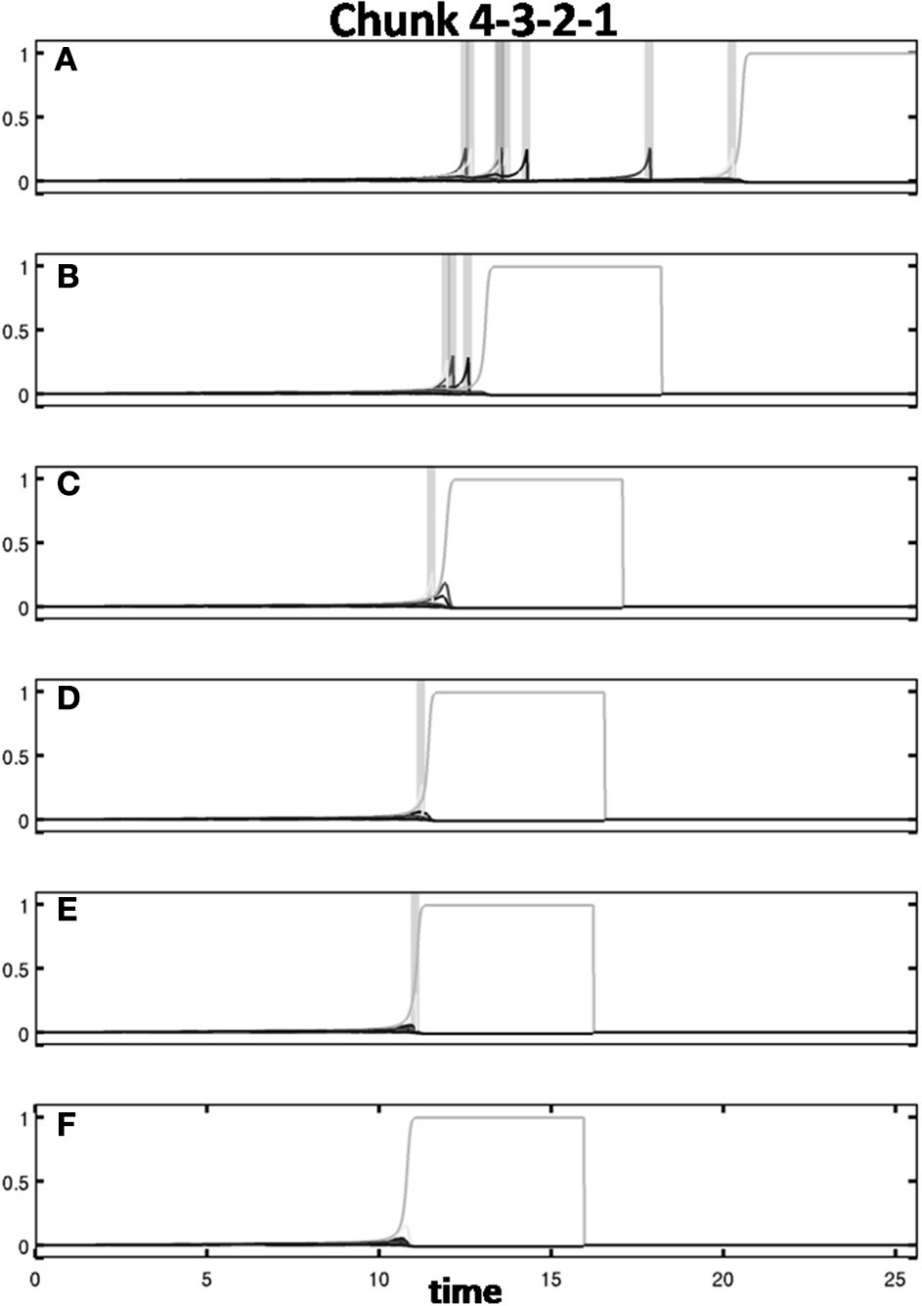
**Activity through time of 4-chunks to the sequence “4-3-2-1” in successive presentation trials (successive rows).** Gray bars denote reset events in which an incorrect list chunk is selected. Once the reset event occurs, the most active cell is shut down, and the remaining list chunks are allowed to compete for activity. On successive trials **(A–F)**, fewer resets occur and the correct list chunk is chosen more quickly. See text for details.

**Figure 14:**
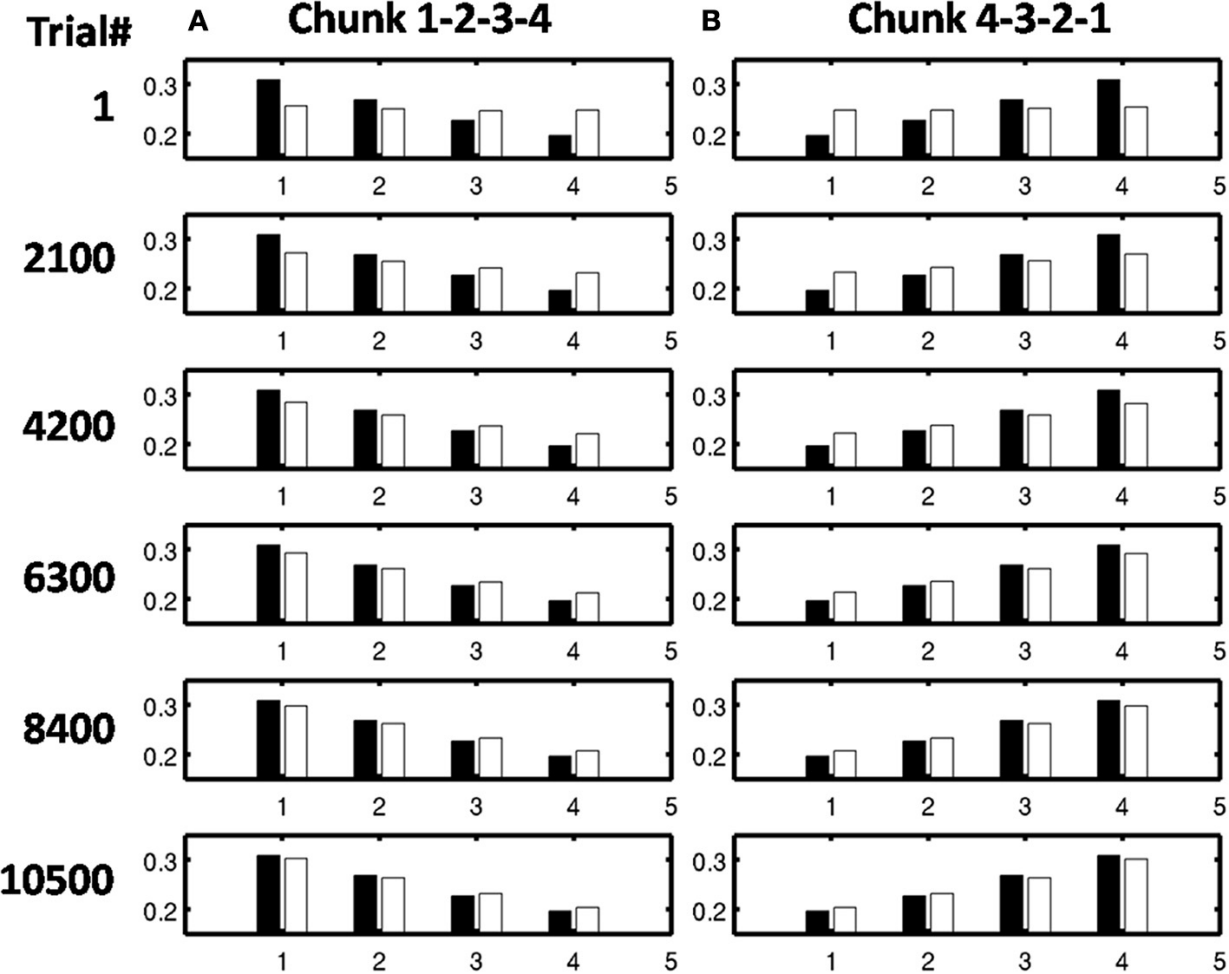
**Convergence over trials to ground truth weights for sequences (A) “1-2-3-4” and (B) “4-3-2-1.”** Learning is much faster than in the unsupervised learning case. By trial 2100 each sequence had been presented 10 times by trial 4200, 20 times; by trial 6300, 30 times; and by trial 8400, 40 times.

Figure 13 shows the response of the Masking Field to presentation of the sequence “4-3-2-1.” The first presentation of this sequence in (Figure 13A) exhibits seven resets in response to the input. By the second presentation of “4-3-2-1,” only three resets occur. In the third, fourth and fifth presentations, only one reset occurs. The correct list chunk is chosen without reset starting with the sixth presentation. Note that the time to select the correct category decreases with each list presentation. Figure 14 illustrates how bottom-up weights converge to the correct pattern through time for list chunks “1-2-3-4” in column 1 and “4-3-2-1” in column 2. Thus, no errors are made long before the weights fully converge to their final weights. This is true also during unsupervised learning with initially balanced weights.

## 8. Model extensions and comparison with other speech and word recognition models

This article models how sequences of items that are stored by an Item-and-Order working memory can learn to selectively activate list chunks in a Masking Field categorization network, as the sequences are presented to the network in real time. The model embodies six new hypotheses that overcome limitations of previous studies. See Section 5. The current article shows how these hypotheses enable real-time unsupervised or supervised learning of list chunks in response to all possible sequences of items.

These new results augment a substantial modeling literature about how Item-and-Order working memories and Masking Fields can be used to explain psychological and neurobiological data about linguistic, spatial, and motor processing of sequentially presented information. Many of these studies assumed that the type of learning which is demonstrated in the current article has occurred. The current simulations therefore help to complete the explanation of these data.

### 8.1. Simulations of data about free recall and sequential copying movements

For example, The LIST PARSE model (Grossberg and Pearson, 2008) was used to simulate human cognitive data about immediate serial recall and immediate, delayed, and continuous distractor free recall of linguistic sequences, as well as monkey neurophysiological data about performance of a sequence of copying movements (Figure 4). LIST PARSE predicts how the laminar circuits of prefrontal cortex (PFC) realize an Item-and-Order working memory and list chunking network, and how interactions with multiple brain regions, notably the ventrolateral prefrontal cortex, dorsolateral prefrontal cortex, motor cortex, cerebellum, and the basal ganglia, can learn to control sequences of movements that can be performed at variable speeds.

### 8.2. Simulating lists with multiple item repetitions: item-order-rank coding

Most implementations of Item-and-Order models utilize a localist representation of list items: single nodes, representing populations of neurons, become active in response to the presentation of a sequence of specific items. In its simplest form, this kind of item representation cannot represent the same item in multiple positions, or ranks, of a list. However, the activity of some neurons in PFC for a given list item is sensitive to the rank of that item within the sequence (Barone and Joseph, 1989; Averbeck et al., 2002, 2003a,b; Mushiake et al., 2006). In addition, human error data in serial recall imply utilization of rank information (e.g., Conrad, 1960; Henson, 2001; Bowman and Wyble, 2007), which some models of serial recall have incorporated (Henson, 1998; Burgess and Hitch, 1999; Brown et al., 2000).

Despite some positive results from rank-based models, Farrell and Lewandowsky (2004) have, as noted in Section 2, shown that latency data from error trials can be best explained by models that use a primacy gradient and self-inhibition (i.e., Item-and-Order models), but not by those that use rank alone. Some Item-and-Order models have incorporated rank information (Bradski et al., 1994; Bohland et al., 2010), but have not explained how rank coding may arise in the brain. Grossberg and Pearson (2008) proposed how an Item-Order-Rank WM can be created in PFC in which rank-order coding is incorporated into an Item-and-Order WM to represent item repeats at arbitrary list positions (e.g., a list like ABACBD) using inputs from numerical representations in the parietal cortex. This prediction built upon the Spatial Number Network, or SpaN, model of Grossberg and Repin (2003) of how numerical representations in inferior parietal cortex may control the ability of animals and humans to estimate and compare numerical quantities. The properties of SpaN model neurons were supported by neurophysiological data of Nieder and Miller (2003, 2004), who also studied the prefrontal projections of these parietal numerical representations. In such an Item-Order-Rank working memory, relative activity still represents the temporal order of the sequences that is stored, but the rank-order sensitivity of the working memory enables the same item to be stored and performed in multiple list positions from the working memory, with later repetitions stored with progressively less activity.

### 8.3. Spatial item-order-rank working memories in prefrontal cortex

The lisTELOS model of Silver et al. (2011) built upon the Grossberg and Pearson (2008) proposal by developing an Item-Order-Rank model of spatial working memory in PFC and its interactions with other brain regions to control the planning, working memory storage, and execution of sequences of saccadic eye movements. In lisTELOS, rank information originates in the model's parietal cortex and projects to the model's rank-sensitive PFC working memory representations. The model predicts and simulates how the supplementary eye fields (SEF) may select saccades that are stored in this PFC working memory. lisTELOS also proposes how SEF may interact with downstream regions such as the frontal eye fields (FEF) during memory-guided sequential saccade tasks, and how the basal ganglia (BG) may control the flow of information through time. Model simulations reproduce behavioral, anatomical and electrophysiological data under multiple experimental paradigms, including visually- and memory-guided single and sequential saccade tasks. Critically, the simulations reproduce behavioral data collected during two SEF microstimulation paradigms (Histed and Miller, 2006; Yang et al., 2008), and show how the seemingly inconsistent findings of these studies about saccade latency can be reconciled by the model.

### 8.4. Are all brain working memories variations of the item-and-order design?

The Grossberg and Pearson (2008) and Silver et al. (2011) articles illustrate how Item-and-Order working memories can quantitatively simulate behavioral and neurophysiological about linguistic, spatial, and motor behaviors. These articles provide converging evidence for the prediction in Grossberg (1978a,b) that all working memories in the brain are variations of the Item-and-Order design in order to enable learning and stable memory of list chunks in response to linguistic, spatial, or motor sequences. Although there is converging evidence for these working memory and list chunking models, they do not, in themselves, form a complete design for brain learning. Some of the missing ingredients are clarified by neural models that provide answers to the following basic question.

### 8.5. What type of stable memory is assured by the LTM invariance principle?

As noted in Section 2, the LTM Invariance Principle insists that, if bottom-up inputs have activated a familiar subset list chunk, such as the word MY, then the arrival of the remaining portion SELF of the novel word MYSELF during the next time interval will not alter the activity pattern of MY in a way that would destabilize the previously learned weights that activate the list chunk of MY. This principle is achieved mathematically by preserving the *relative activities*, or ratios, between working memory activities as new items are presented through time.

The importance of relative activities arises from the fact that working memory outputs are multiplied by adaptive weights and added before activating list chunks. This combination of multiplication and addition is called a dot product, or inner product. The term ∑_*i* ∈ *J*_ *x*_*i*_*Z*_*i*_*W*_*ij*_ in Equation (6) defines such a dot product whereby the habituative signals *x*_*i*_*Z*_*i*_ are multiplied by the adaptive weights *W*_*ij*_. Learning tunes the vector of adaptive weights to make them more parallel to the activities that they multiply, thereby making the total input to their target cell larger.

Preserving the relative activities of stored items in the working memory when new inputs occur ensures that these activities remain parallel to their previously learned weight vectors. Preserving relative activities thus does not force unlearning of the previously learned weight vectors. This kind of stability does not, however, protect previously learned weights from being catastrophically recoded in response to sufficiently complex input environments, as demonstrated in Grossberg (1976b) and then proved for cyclically repeating lists of just length four in Carpenter and Grossberg (1987). In such environments, the recoding of adaptive weights can be driven by supersets of the inputs from which they originally learned. Examples of catastrophic forgetting led Grossberg (1976a, 1980) to pose the *stability-plasticity dilemma*, or how a brain, or machine, can learn quickly without also be forced to experience catastrophic forgetting.

### 8.6. Attention, resonance, learning, and consciousness

Item-and-Order working memory and Masking Field list chunking networks were first derived as part of larger neural architectures that were designed to solve the stability-plasticity dilemma. Such architectures include both bottom-up interactions, such as the adaptive filter ∑_*i* ∈ *J*_*x*_*i*_*Z*_*i*_*W*_*ij*_ in Equation (6), and recurrent horizontal interactions, such as the excitatory feedback term *D*|*J*|*f*(*c*_*j*_) in Equation (6) that helps to choose a winning list chunk. But they do not include top-down interactions from a higher processing level to a lower one.

Speech and language data motivated the development of neural models that include bottom-up, horizontal, and top-down interactions. These models propose answers to the following questions: What is the neural representation of a speech code as it evolves in time? How do listeners integrate temporally distributed phonemic information across hundreds of milliseconds, even backwards in time, into coherent representations of phonemes, syllables and words? How do these representations give rise to context-sensitive speech percepts in the correct temporal order, despite such backwards effects? How do these representations learn from experience in a way that solves the stability-plasticity dilemma?

The models answer these questions by hypothesizing that conscious auditory and speech percepts are emergent properties that arise from *resonant* states of the brain (Grossberg, 1978a, 1986, 2003; Grossberg et al., 1997; Grossberg and Myers, 2000; Grossberg and Kazerounian, 2011). Such resonant states develop when bottom-up signals that are activated by, and learn from, acoustic stimuli interact with top-down expectations, or prototypes, that are also learned from prior experience. Each top-down expectation controls a matching process that selects bottom-up features that are consistent with its learned prototype, while inhibiting those that are not. In this way, an attentional focus starts to develop that concentrates activation on those feature clusters that are deemed important, based on past experience. The attended feature clusters, in turn, reactivate the cycle of bottom-up and top-down signal exchange.

This reciprocal exchange of bottom-up and top-down signals causes the selected cells to resonate with amplified and synchronized activities. Such a resonance binds the attended features together into a coherent brain state. Resonant states, rather than the activations that are due to bottom-up processing alone, are predicted to drive fast learning whose memories are dynamically stabilized by top-down attentive matching, hence the name *Adaptive* Resonance Theory for this class of models. It has been mathematically proved that the properties of this top-down attentive matching process, called the ART Matching Rule, are necessary to enable fast learning to occur in response to a dense non-stationary input sequences without causing catastrophic forgetting (Carpenter and Grossberg, 1987, 1991). What is learned are prototypes derived from the attended activation patterns during resonant events. ART also predicts that “all conscious events are resonant events” and thus mechanistically describes a relationship between attention and consciousness.

### 8.7. Why is the immediate memory span longer than the transient memory span?

As noted in Section 2, the transient memory span was defined in Grossberg (1978a,b) as the longest list length for which a working memory can store a primacy gradient in response to only bottom-up inputs. The immediate memory span was predicted to be the result of combining bottom-up and top-down inputs to the working memory, where the top-down inputs are activated by the list chunks that themselves get activated by bottom-up inputs from the working memory. These top-down inputs are learned expectations that have been stored in LTM. In brief, the transient memory span is due primarily to storage in STM, whereas the immediate memory span is due to a combination of STM and LTM.

Grossberg (1978a) proved that read-out of list chunk top-down expectations from LTM into working memory STM generates an extended primacy gradient in working memory, longer than the transient memory span. Thus, ART top-down feedback to an Item-and-Order working memory leads to an immediate memory span that is longer than the transient memory span. Because the immediate memory span is estimated at seven, as in G.A. Miller's Magical Number Seven (Miller, 1956), Grossberg (1978a) predicted that the transient memory span would be around four. Cowan (2001) reviewed subsequent data that support the existence of a 4 ± 1 working memory capacity limit when learning and grouping influences are minimized. Indeed, long-term memory (LTM) does bias working memory toward more primacy dominance (e.g., Knoedler et al., 1999), and its influence can be difficult to limit. Cowan (2001) reviewed proposals for limiting LTM influence, such as using novel sequence orderings of well-learned items that are difficult to group. This property exists in addition to the ability of a Masking Field to represent 7 ± 2 list chunks, even when the length of the largest learned chunks varies due to learning.

### 8.8. Magical number seven and word superiority

A word length effect in word superiority studies also follows from the self-similarity property. This word length effect was discovered by Samuel et al. (1982, 1983) who showed that a letter is progressively better recognized when it is embedded in longer words of lengths from 1 to 4. The word length effect is partially explained by self-similarity, since larger list chunks are more potent and predictive than smaller list chunks in a Masking Field. However, self-similarity implies that the list chunk of a familiar multi-letter word can *inhibit* the list chunk of a familiar letter, which seems to contradict the property that the word can *facilitate* perception of its constituent letters, which is the main result of word superiority studies.

This problem is resolved in ART systems with item and list chunk processing levels. In particular, although chunks that represent lists of multiple lengths compete within the Masking Field that categorizes list chunks, the top-down expectations from the list chunk level to the item level are excitatory. By self-similarity, list chunks that represent longer words generate larger recurrent inhibitory signals *and* top-down excitatory signals. List chunks that represent longer lists will therefore send larger top-down excitatory priming signals to the item chunk level, thereby explaining both how the Magical Number Seven can arise due to asymmetric inhibition among list chunks, and how a word length effect in word superiority can arise due to greater top-down excitation from longer list chunks to the item chunks that activate them. Both of these properties, in turn, further support the ART prediction that Item-and-Order working memories that represent item chunks and list chunks are the units of processing (Grossberg, 1978a, 1984), not the phonemes, letters, and words that were used in the Interactive Activation Model (McClelland and Rumelhart, 1981).

### 8.9. Laminar cortical model of conscious speech perception

**Figure 15:**
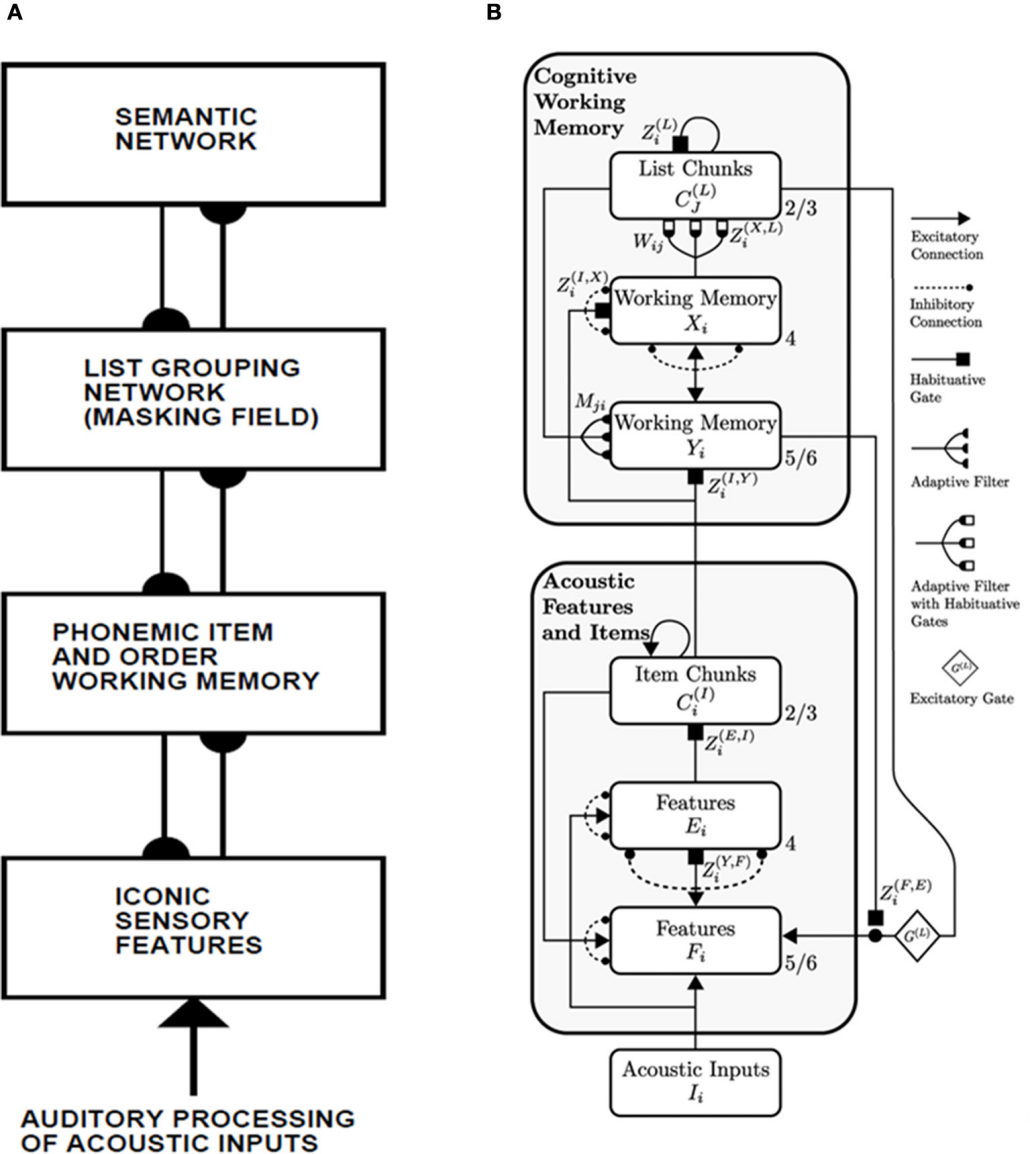
**(A)** ARTWORD processing levels for speech perception. (Reprinted with permission from Grossberg and Myers, 2000). **(B)** cARTWORD laminar cortical model for conscious speech perception. (Reprinted with permission from Grossberg and Kazerounian, 2011).

An Item-and-Order working memory and Masking Field, together with learned top-down expectations, are all incorporated into the ARTWORD model of how speech is categorized (Figure 15A; Grossberg and Myers, 2000). The Conscious ARTWORD, or cARTWORD, model (Figure 15B; Grossberg and Kazerounian, 2011) further develops the concepts and mechanisms of ARTWORD to simulate how the laminar circuits of several cortical regions interact to give rise to conscious speech percepts. Lower levels in the cARTWORD hierarchy consist of peripheral auditory neurons that are responsible for early acoustic processing. As acoustic inputs arrive over time, these peripheral auditory neurons send signals to higher-level neurons that encode feature detectors. A pattern of activation across these feature detectors then activates a compressed item chunk representation. A sequence of item chunks is temporarily stored in working memory as a temporal succession of sounds occurs. As in all Item-and-Order working memories, the speech working memory transforms the temporal sequence of item activations into an evolving spatial pattern of activity that represents both the items that were presented, as well as the temporal order in which they occurred. The activity patterns across the Item-and-Order working memory activate Masking Field list chunks via an adaptive filter. These list chunks may represent, for example, phonemes, syllables, or words.

The model of chunk learning in Figure 1 was motivated by, and can be embedded into, the working memory and list chunk processing levels of cARTWORD. The current article shows, in effect, how learning by the adaptive weights in the adaptive filter converts Masking Field nodes into list chunks that selectively respond to a particular temporal sequence of items.

### 8.10. Features, items, and list chunks instead of phonemes, letters, and words

The classical work of Miller (1956) on the Magical Number Seven showed that one of the important functional units in speech and language is abstract, namely the “chunk.” Chunks can be composed of multiple types of acoustic inputs that vary in size. The current model extends that insight to the entire hierarchy of feature, item (chunk), and list chunk processing levels, and embodies it in the cARTWORD laminar cortical model.

One of the key insights of models like ARTWORD and cARTWORD has thus been to extend the classical work of Miller (1956) on (list) chunks to redefine the functional units that are proposed to exist at successive levels of the brain's speech and language hierarchy of processing stages. In particular, instead of stages in the hierarchy that process phonemes, letters, and words (e.g., McClelland and Rumelhart, 1981), the stages in these models were posited to represent distributed features, items, and list chunks (Grossberg, 1978b, 1984, 1986), and were used to explain data about word superiority effect, list length effect, and related speech phenomena that were not explicable by alternative processing levels. Experiments that tested these processing levels with positive results have been described in Vitevitch and Luce (1999).

Both the item chunk and list chunk stages can represent phonemes and letters, and the list chunk stage can represent phonemes, syllables, and words. For example, a familiar letter that is also a word, such as A and I, and a familiar letter that is not a word, such as E and F, have both item and list chunk representations because they are both familiar. In other words, all familiar letters and words are lists. In a hierarchy of letter and word stages, in contrast, only letters that are words would have both a letter and a word representation. One might therefore expect that letters that are words, but not letters that are not words, would have the benefit of excitatory top-down priming by their word representations, and would thus be recognized more easily than letters that are not words. This difference is not, however, found in word recognition experiments (Wheeler, 1970). Because sequences of items can represent phonemes, syllables, or words, any of these groupings can, in principle, be represented by list chunks, depending upon the temporal context in which they occur. In a hierarchy of phoneme, letter, and word stages, such co-existence of different types of linguistic representations at a single processing stage does not occur.

When the feature, item chunk, and list chunk levels were first introduced, one of their predicted properties was that they could be learned during real-time processing of auditory sequences, but that the alternative levels of phonemes, letters, and words could not. The current article supports this prediction with simulations of list chunk learning in real time.

### 8.11. Contextual disambiguation using top-down attentive matching: phonemic restoration

Many more data can be naturally explained with the addition of learned top-down expectations that are attentively matched against bottom-up feature patterns. One important example is phonemic restoration, which was first described by Warren (1970), Warren and Warren (1970). For example, suppose that a phoneme such as the /s/ in the word “legislature” is excised, and replaced by broadband noise. Then the excised phoneme is perceived by listeners as being present and intact in the stimulus. When the phoneme is removed and simply replaced by silence, then the silence gap is perceived and no such restoration occurs.

The phoneme that is restored can also depend on acoustic signals that arrive after the broadband noise is presented. For example, Warren and Sherman (1974) showed that the phoneme to be restored could be determined solely by subsequent context due to acoustic input arriving after the deleted phoneme. This study considered two words, “delivery” and “deliberation,” which are contextually neutral until the /v/ or /b/. Before presentation of /v/ or /b/, the initial portions of the two words, “deli” are virtually indistinguishable and do not contain sufficient co-articulatory information to predict whether /v/ or /b/ will follow. After presentation of “deli^*^” (where ^*^ denotes noise), this speech segment was then followed by either “ery” or “eration.” Presentation of “ery” resulted in the perceptual restoration of the phoneme /v/, whereas presentation of “eration” in the restoration of the phoneme /b/. The critical question arising from this study regards how future acoustical events interact with past stimuli to form conscious percepts in a manner whereby the disambiguating cue (“y” or “ation” in “delivery” and “deliberation,” respectively) can influence earlier stimuli, and can do so without destructive interference of intervening portions such as “er.”

Why is the noise in “deli-noise-[ery/eration]” not heard before the last portion of the word is even presented? This may be explained by the fact that, if the resonance has not developed fully before the last portion of the word is presented, then this portion can influence the top-down expectations that determine the conscious percept. How does such an expectation convert the noise in “deli-noise-[ery/eration]” into a percept of [/v/-/b/]? This occurs due to the top-down matching process that selects expected feature clusters for attentive processing while suppressing unexpected ones. In the “deli-noise-[ery/eration]” example, spectral components of the noise are suppressed that are not part of the expected consonant sound. As noted above, it has been mathematically proved that the properties of this top-down attentive matching process, called the ART Matching Rule, are necessary to enable fast learning to occur without causing catastrophic forgetting (Carpenter and Grossberg, 1987, 1991). Thus, phonemic restoration illustrates attentive matching processes that enable speech and language to be learned quickly without forcing catastrophic forgetting of previously learned memories.

### 8.12. Why the LTM invariance principle and the ART matching rule are both needed

The phenomenon of phonemic restoration, and the cARTWORD simulation of it, clarifies that the resonance that supports the conscious percept of a word typically occurs only *after* all the items that form the word have been entered into working memory. Thus, the stabilizing effect on learned memories of the ART Matching Rule is not available to deal with the possible destabilizing effect on memory that would have been caused if, for example, the processing of SELF after MY during the presentation of MYSELF changed the ratios of activities of the items that represent MY in working memory. Thus, the LTM Invariance Principle and the ART Matching Rule each contribute to memory stability, but in different ways.

### 8.13. Alternative speech models: TRACE, MERGE, and TISK

Alternative neural models have attempted to show how sequences, and, in particular, sequences of speech sounds, may be represented in the brain. By ignoring neurophysiological constraints however, and by not considering how such sequential representations may be learned, these models typically fail to adequately account for the form those sequence representations take in the brain.

One of the earliest and most prominent of these models is the TRACE model of speech perception (McClelland and Elman, 1986). The TRACE model attempts to account for bottom-up and top-down interactions between phonemes (the constituent parts of speech sequences) and words (the sequences themselves). However, this model does not deal with the sequential and temporal nature of sequences. Instead, it duplicates the representation of every phoneme and word at each time step. This hypothesis faces fundamental computational problems, including: (a) replicating speech tokens in multiple time slices causes a combinatorial explosion during the representation of realistic speech and language; and (b) such a representation would seem to make learning of a self-organizing time-invariant speech understanding system impossible. In addition, there are biological problems: (c) replicating speech representations at each time has no neuroanatomical or neurophysiological support and is, in fact, incompatible with neurophysiological data about how items are stored in working memory (e.g., Figure 4); (d) the replication process represents silence as a separate node, a hypothesis that is unable to explain the many data that demonstrate contextual effects on the perception of silence during speech perception (e.g., Repp et al., 1978; Repp, 1980). In contrast, many of these contextual effects have been quantitatively simulated by the PHONET, ARTPHONE, ARTWORD, and cARTWORD models (e.g., Grossberg et al., 1997; Boardman et al., 1999; Grossberg and Myers, 2000; Grossberg and Kazerounian, 2011).

Models that followed TRACE include the MERGE model (Norris et al., 2000), which itself built off the SHORTLIST model (Norris, 1994). While overcoming some of the limitations of TRACE (e.g., node/weight reduplication), they were not without problems of their own. For example, MERGE has a feedforward architecture that does not include any top-down feedback. It thus cannot explain the voluminous data about top-down attentive priming effects on language meaning, cannot explain how speech and language self-organize so quickly without experiencing catastrophic forgetting, and cannot explain how the brain can complete speech representations that are partially occluded by noise, as again the ARTWORD and cARTWORD models have done. No less importantly, the MERGE model requires “on-the-fly” wiring between the lexical, phoneme, and phoneme decision nodes, meaning that the architecture is task-dependent, and is unable to learn these representations.

Most recently, the Time Invariant String Kernel (TISK) model of word recognition (Hannagan et al., 2013) has attempted to maintain some of the desirable properties of the TRACE model, while eliminating its massive reduplication of nodes and weights in order to represent time. Before comparing this model with the current model, it will be noted that the criticisms that Hannagan et al. have made about cARTWORD are incorrect.

They write in their Section 4.3: “Another approach is the cARTWORD model of Grossberg and Kazerounian (2011), where activity gradients specific to particular sequences can differentiate orderings of the same elements (e.g., ABC vs. ACB, BAC, etc.). However, this mechanism cannot represent sequences with repeated elements (for example, it cannot distinguish ABCB from ABC, as the second B would simply provide further support for B rather than a second B event), which makes it incapable of representing nearly one third of English lemmas.” The cARTWORD simulations of Grossberg and Kazerounian (2011) did not include repeated elements because that was not the explanatory goal of that article, just as it is not the focus of the current article. The discussion above about how Item-Order-Rank working memories and learned chunks can easily represent repeated elements, and how they can explain neurobiological data about how this is accomplished in the brain (e.g., Grossberg and Pearson, 2008; Silver et al., 2011), shows that this claim is incorrect.

Hannagan et al. (2013) also erroneously claim that “it is premature to compare this approach to models like TRACE, since it has been applied to a single phenomenon (phoneme restoration).” cARTWORD is, however, a natural development of a family of ART-based speech and language models dating back to the 1970s; e.g., PHONET, ARTPHONE, and ARTWORD, as well as SPINET and ARTSTREAM (Cohen et al., 1995; Grossberg et al., 2004). These models have explained and simulated a wide range of data about auditory streaming, speech perception, and word recognition. cARTWORD, in particular, showed how ARTWORD concepts and mechanisms could be extended to show how specializations of the laminar cortical circuits that have elsewhere been used to explain vision and cognition data (e.g., Raizada and Grossberg, 2003; Grossberg and Pearson, 2008; Grossberg and Versace, 2008) can be specialized to also explain speech data. Thus, cARTWORD is an example of a much broader theory of neocortical function that currently has an unrivaled explanatory and predictive range. The ART-based theory of speech and language that has been getting self-consistently developed over several decades, of which cARTWORD is just one example, has, in fact, explained and predicted many data about speech and word recognition that TISK, TRACE, and MERGE have not explained.

The final claim of Hannagan et al. (2013) is about “the supposed failures of TRACE to account for phoneme restoration [which are] the result of flawed simulations, not a problem of TRACE.” In particular, Hannagan et al. (2013) asserted that simulations showing how TRACE could simulate phonemic restoration were then being submitted by Magnuson for publication. A reviewer of Grossberg and Kazerounian (2011) made a similar claim about the jTRACE model and included simulations to illustrate that claim. The Discussion section of Grossberg and Kazerounian (2011) responded with their own simulations (see their Figure 9) showing that there are serious conceptual and data-explanatory problems with the jTRACE simulations.

The repeated time slices in TRACE is eliminated in TISK primarily for expedience because, as is clear from the combinatorial explosion inherent in TRACE, it takes too long to simulate TRACE with realistically sized lexicons. Some reduplication is avoided in the TISK model by making use of string kernels (Hofmann et al., 2008), whereby a symbol sequence, or string, is represented in a high-dimensional space of symbol combinations. For example, a string such as “DOG,” would be represented by a combination of its ordered diphones, /da/, /dg/, and /ag/, whereas a word such as “GOD” would be represented by /ga/, /gd/, /ad/. By only representing the forward ordered diphones in the word, the model is able to distinguish between words comprised of the same phonemes, but in different orderings. The authors note that: “the use of time-specific nodes at this level is a matter of computational convenience without theoretical commitment or consequence; these nodes provide a computationally expedient way to pass sequences of phonemic inputs to the model, and could conceivably be replaced by a single bank of input nodes (but this would require other additions to the model to allow inputs to be ‘scheduled’ over time).”

This sequence representational hypothesis is, however, a highly constraining “theoretical commitment,” since the TISK model, just like the TRACE model, sidesteps how speech inputs are processed in time, how they activate time-variant phoneme representations, and how a self-organizing learning process can learn unitized phoneme, syllable, and word representations from time-varying acoustic inputs. Given the critical role of time-varying contextual effects on the consciously heard phonetics and meaning of speech and language, trying to represent them without temporal dynamics is a program that is fraught with theoretical dangers, since if their basic representation of speech is wrong, then all conclusions drawn from it collapse.

In addition, just as with TRACE, many time-dependent contextual effects on consciously heard speech cannot be understood within TISK. For example, if the ordered diphone /ba/ is a primitive of the model, then it seems impossible to explain, say, how /ba/ can sound like /wa/ when the duration of the vowel /a/ is suitably varied (Miller and Liberman, 1979; Schwab et al., 1981), as has been quantitatively simulated by the PHONET model variant of ARTWORD (Boardman et al., 1999).

Another problem with TISK is its absence of feedback connections. As the authors note: “We acknowledge that without feedback, TISK will not be able to simulate many top–down phenomena readily simulated in TRACE. Future research with TISK will explore the impact of feedback connections.” Even if, however, feedback connections were added to TISK, it is not clear how it would clarify the conscious percepts of listeners, particularly in cases where feedback is necessary, as in the Ganong effect and phonemic restoration. If feedback is from the lexical layer to the nphone (diphone) layer, the percept cannot correspond to activations of the diphones. As the example of the word “DOG” illustrates, the active diphone /dg/ is never heard. If feedback is tiered, such that there is lexical feedback to the diphone layer, and diphone feedback to the phoneme layer, a diphone such as /da/ in “DOG,” would activate phoneme representations for /d/ and /a/ at all time-variant positions. As such, activation in the phoneme layer is equally incapable of corresponding to a listener's percept. Lastly, there is no mechanism in TISK whereby feedback from the lexical to phonemic layer can coordinate the temporal activations, in correct order, such that the ensuing resonant phonemic activations correspond to a listener's conscious speech percepts.

In contrast, the top-down attentive feedback in all ART-based models of learning and recognition has the form of a top-down, modulatory on-center, off-surround network that was mathematically proved to enable fast learning without catastrophic forgetting (e.g., Carpenter and Grossberg, 1987), that has been used to explain and simulate many data about how attention and category learning work, and has been supported by a large number of subsequent psychological and neurobiological data. See Grossberg (2012) for a review.

## 9. Future directions

Future work within the current theoretical framework can profitably show how a Masking Field's recurrent connections can robustly develop through learning in response to both endogenously active inputs from its working memory during a critical period before environmentally-driven learning begins, and to environmentally-driven inputs during the postnatal period. Such a study would clarify how the balance between and selectivity and predictivity of Masking Field cells develops. In addition, an embedding of the list chunk learning process into a cortical hierarchy that is capable of resonant self-stabilizing learning, such as the cARTWORD architecture depicted in Figure 5B, remains to be carried out in response to a realistic speech environment. When these additional contributions are completed, the cARTWORD architecture will be ready to offer a radically different approach to applications in speech recognition technology than are currently available.

### Conflict of interest statement

The authors declare that the research was conducted in the absence of any commercial or financial relationships that could be construed as a potential conflict of interest.

## References

[B1] Abbott L. F. Sen K. Varela J. A. Nelson S. B. (1997). Synaptic depression and cortical gain control. Science 275, 220–222 10.1126/science.275.5297.221 8985017

[B5] Averbeck B. B. Chafee M. V. Crowe D. A. Georgopoulos A. P. (2002). Parallel processing of serial movements in prefrontal cortex. Proc. Natl. Acad. Sci. U.S.A. 99, 13172–13177 10.1073/pnas.162485599 12242330 PMC130605

[B6] Averbeck B. B. Crowe D. A. Chafee M. V. Georgopoulos A. P. (2003a). Neural activity in prefrontal cortex during copying geometrical shapes. I. Single cells encode shape, sequence, and metric parameters. Exp. Brain Res. 150, 127–141 10.1007/s00221-003-1416-6 12669170

[B7] Averbeck B. B. Crowe D. A. Chafee M. V. Georgopoulos A. P. (2003b). Neural activity in prefrontal cortex during copying geometrical shapes. II. Decoding shape segments from neural ensembles. Exp. Brain Res. 150, 142–153 10.1007/s00221-003-1417-5 12669171

[B8] Barone P. Joseph J. (1989). Prefrontal cortex and spatial sequencing in macaque monkey. Exp. Brain Res. 78, 447–464 10.1007/BF00230234 2612591

[B9] Berzhanskaya J. Grossberg S. Mingolla E. (2007). Laminar cortical dynamics of visual form and motion interactions during coherent object motion perception. Spat. Vis. 20, 337–395 10.1163/156856807780919000 17594799

[B10] Boardman I. Bullock D. (1991). A neural network model of serial order recall from short-term memory, in International Joint Conference on Neural Networks (Seattle, WA), 879–884 10.1109/IJCNN.1991.155450

[B11] Boardman I. Grossberg S. Myers C. Cohen M. (1999). Neural dynamics of perceptual order and context effects for variable-rate speech syllables. Percept. Psychophys. 6, 1477–1500 10.3758/BF03213112 10598464

[B12] Bohland J. Bullock D. Guenther F. (2010). Neural representations and mechanisms for the performance of simple speech sequences. J. Cogn. Neurosci. 22, 1504–1529 10.1162/jocn.2009.21306 19583476 PMC2937837

[B13] Bowman H. Wyble B. (2007). The simultaneous type, serial token model of temporal attention and working memory. Psychol. Rev. 114, 38–70 10.1037/0033-295X.114.1.38 17227181

[B14] Bradski G. Carpenter G. A. Grossberg S. (1994). Store working memory networks for storage and recall of arbitrary temporal sequences. Biol. Cybern. 71, 468–480 10.1007/BF00198465

[B15] Brown G. Preece T. Hulme C. (2000). Oscillator-based memory for serial order. Psychol. Rev. 107, 127–181 10.1037/0033-295X.107.1.127 10687405

[B16] Bullock D. Rhodes B. (2003). Competitive queuing for planning and serial performance, in Handbook of Brain Theory and Neural Networks, ed Arbib M. (Cambridge, MA: MIT Press), 241–244

[B17] Burgess N. Hitch G. (1999). Memory for serial order: a network model of the phonological loop and its timing. Psychol. Rev. 106, 551–581 10.1037/0033-295X.106.3.551

[B18] Carpenter G. A. Grossberg S. (1987). A massively parallel architecture for a self-organizing neural pattern recognition machine. Comput. Vis. Graph. Image Process. 37, 54–115 10.1016/S0734-189X(87)80014-212662581

[B20] Carpenter G. A. Grossberg S. (1991). Pattern Recognition by Self-Organizing Neural Networks. Cambridge, MA: MIT Press

[B23] Cohen M. Grossberg S. (1986). Neural dynamics of speech and language coding: developmental programs, perceptual grouping, and competition for short-term memory. Hum. Neurobiol. 5, 1–22 3516940

[B24] Cohen M. Grossberg S. (1987). Masking fields: a massively parallel neural architecture for learning, recognizing, and predicting multiple groupings of patterned data. Appl. Opt. 26, 1866–1891 10.1364/AO.26.001866 20454417

[B22] Cohen M. A. Grossberg S. Wyse L. L. (1995). A spectral network model of pitch perception. J. Acoust. Soc. Am. 98, 862–879 10.1121/1.413512 7642825

[B25] Conrad R. (1960). Serial order intrusions in immediate memory. Br. J. Psychol. 51, 45 10.1111/j.2044-8295.1960.tb00723.x 13811577

[B26] Conrad R. (1965). Order error in immediate recall of sequences. J. Verb. Learn. Verb. Behav. 4, 161–169 10.1016/S0022-5371(65)80015-9

[B27] Cowan N. (2001). The magical number 4 in short-term memory: a reconsideration of mental storage capacity. Behav. Brain Sci. 24, 87–185 10.1017/S0140525X01003922 11515286

[B28] Douglas R. J. Koch C. Mahowald M. Martin K. A. C. Suarez H. H. (1995). Recurrent excitation in neocortical circuits. Science 269, 981–985 10.1126/science.7638624 7638624

[B29] Dranias M. Grossberg S. Bullock D. (2008). Dopaminergic and non-dopaminergic value systems in conditioning and outcome-specific revaluation. Brain Res. 1238, 239–287 10.1016/j.brainres.2008.07.013 18674518

[B30] Ebbinghaus H. (1913). On Memory: A Contribution to Experimental Psychology (Transl. eds Ruger H. Bussenius C.). New York, NY: Teachers College

[B31] Farrell S. Lewandowsky S. (2004). Modeling transposition latencies: constraints for theories of serial order memory. J. Mem. Lang. 51, 115–135 10.1016/j.jml.2004.03.007

[B32] Fazl A. Grossberg S. Mingolla E. (2009). View-invariant object category learning, recognition, and search: how spatial and object attention are coordinated using surface-based attentional shrouds. Cogn. Psychol. 58, 1–48 10.1016/j.cogpsych.2008.05.001 18653176

[B33] Francis G. Grossberg S. (1996). Cortical dynamics of boundary segmentation and reset: persistence, afterimages, and residual traces. Perception 35, 543–567 10.1068/p250543 8865297

[B34] Francis G. Grossberg S. Mingolla E. (1994). Cortical dynamics of feature binding and reset: control of visual persistence. Vision Res. 34, 1089–1104 10.1016/0042-6989(94)90012-4 8160417

[B35] Gaudiano P. Grossberg S. (1991). Vector associative maps: unsupervised real-time error-based learning and control of movement trajectories. Neural Netw. 4, 493–504 10.1016/0893-6080(91)90002-M

[B36] Glanzer M. Cunitz A. R. (1966). Two storage mechanisms in free recall. J. Verb. Learn. Verb. Behav. 5, 351–360 10.1016/S0022-5371(66)80044-012364533

[B37] Grossberg S. (1968). Some physiological and biochemical consequences of psychological postulates. Proc. Natl. Acad. Sci. U.S.A. 60, 758–765 10.1073/pnas.60.3.758 5243922 PMC225115

[B38] Grossberg S. (1969). On the serial learning of lists. Math. Biosci. 4, 201–253 10.1016/0025-5564(69)90014-5

[B39] Grossberg S. (1972). A neural theory of punishment and avoidance, II: quantitative theory. Math. Biosci. 15, 232–285

[B40] Grossberg S. (1973). Contour enhancement, short-term memory, and constancies in reverberating neural networks. Stud. App. Math. 52, 213–257

[B41] Grossberg S. (1974). Classical and instrumental learning by neural networks, in Progress Theoretical Biology, eds Rosen R. Snell F. (New York, NY: Academic Press), 51–141 10.1016/B978-0-12-543103-3.50009-2

[B42] Grossberg S. (1976a). Adaptive pattern classification and universal recoding, I: parallel development and coding of neural feature detectors. Biol. Cybern. 23, 121–134 10.1007/BF00344744 974165

[B43] Grossberg S. (1976b). Adaptive pattern classification and universal recoding, II: feedback, expectation, olfaction, and illusions. Biol. Cybern. 23, 187–202 963125 10.1007/BF00340335

[B44] Grossberg S. (1978a). A theory of human memory: self-organization and performance of sensory-motor codes, maps, and plans, in Progress in Theoretical Biology, Vol. 5, eds Rosen R. Snell F. (New York, NY: Academic Press), 233–374 10.1016/B978-0-12-543105-7.50013-0

[B45] Grossberg S. (1978b). Behavioral contrast in short-term memory: serial binary memory models or parallel continuous memory models? J. Math. Psychol. 3, 199–219 10.1016/0022-2496(78)90016-0

[B46] Grossberg S. (1980). How does a brain build a cognitive code? Psychol. Rev. 87, 1–51 10.1037/0033-295X.87.1.1 7375607

[B47] Grossberg S. (1984). Unitization, automaticity, temporal order, and word recognition. Cogn. Brain Theory 7, 263–283

[B48] Grossberg S. (1986). The adaptive self-organization of serial order in behavior: speech, language, and motor control, in Pattern Recognition by Humans and Machines, Speech Perception Vol. 1, eds Schwab E. C. Nusbaum H. C. (New York, NY: Academic Press), 187–294

[B49] Grossberg S. (1999). How does the cerebral cortex work? Learning, attention and grouping by the laminar circuits of visual cortex. Spat. Vis. 12, 163–186 10.1163/156856899X00102 10221426

[B50] Grossberg S. (2003). Resonant neural dynamics of speech perception. J. Phonetics 31, 423–445 10.1016/S0095-4470(03)00051-2

[B51] Grossberg S. (2007). Consciousness CLEARS the mind. Neural Netw. 20, 1040–1053 10.1016/j.neunet.2007.09.014 17964756

[B112] Grossberg S. (2012). Adaptve resonance theory: how a brain learns to consciously attend, learn, and recognize a changing world. Neural Netw. 37, 1–47 10.1016/j.neunet.2012.09.017 23149242

[B52] Grossberg S. Boardman I. Cohen M. (1997). Neural dynamics of variable-rate speech categorization. J. Exp. Psychol. Hum. Percept. Perform. 23, 418–503 10.1037/0096-1523.23.2.481 9104006

[B53] Grossberg S. Govindarajan K. K. Wyse L. L. Cohen M. A. (2004). ARTSTREAM: a neural network model of auditory scene analysis and source segregation. Neural Netw. 17, 511–536 10.1016/j.neunet.2003.10.002 15109681

[B54] Grossberg S. Kazerounian S. (2011). Laminar cortical dynamics of conscious speech perception: a neural model of phonemic restoration using subsequent context in noise. J. Acoust. Soc. Am. 130, 440–460 10.1121/1.3589258 21786911

[B56] Grossberg S. Mingolla E. (1985). Neural dynamics of perceptual grouping: textures, boundaries, and emergent segmentations. Percept. Psychophys. 38, 141171 10.3758/BF03198851 4088806

[B57] Grossberg S. Myers C. (2000). The resonant dynamics of speech perception: interword integration and duration-dependent backward effects. Psychol. Rev. 107, 737–767 10.1037/0033-295X.107.4.735 11089405

[B58] Grossberg S. Pearson L. (2008). Laminar cortical dynamics of cognitive and motor working memory, sequence learning and performance: toward a unified theory of how the cerebral cortex works. Psychol. Rev. 115, 677–732 10.1037/a0012618 18729596

[B59] Grossberg S. Pilly P. K. (2012). How entorhinal grid cells may learn multiple spatial scales from a dorsoventral gradient of cell response rates in a self-organizing map. PLoS Comput. Biol. 8:1002648 10.1371/journal.pcbi.1002648 23055909 PMC3464193

[B60] Grossberg S. Pilly P. K. (2013). Coordinated learning of grid cell and place cell spatial and temporal properties: multiple scales, attention, and oscillations. Philos. Trans. R. Soc. Lond. 369:20120524 10.1098/rstb.2012.0524 24366136 PMC3866446

[B61] Grossberg S. Repin D. (2003). A neural model of how the brain represents and compares multi-digit numbers: spatial and categorical processes. Neural Netw. 16, 1107–1140 10.1016/S0893-6080(03)00193-X 13678618

[B62] Grossberg S. Seitz A. (2003). Laminar development of receptive fields, maps, and columns in visual cortex: the coordinating role of the subplate. Cereb. Cortex 13, 852–863 10.1093/cercor/13.8.852 12853372

[B63] Grossberg S. Swaminathan G. (2004). A laminar cortical model for 3D perception of slanted and curved surfaces and of 2D images: development, attention and bistability. Vision Res. 44, 1147–1187 10.1016/j.visres.2003.12.009 15050817

[B64] Grossberg S. Todorovic D. (1988). Neural dynamics of 1-D and 2-D brightness perception: a unified model of classical and recent phenomena. Percept. Psychophys. 43, 241–277 10.3758/BF03207869 3347487

[B65] Grossberg S. Versace M. (2008). Spikes, synchrony, and attentive learning by laminar thalamocortical circuits. Brain Res. 1218, 278–312 10.1016/j.brainres.2008.04.024 18533136

[B66] Grossberg S. Williamson J. R. (2001). A neural model of how horizontal and interlaminar connections of visual cortex develop into adult circuits that carry out perceptual groupings and learning. Cereb. Cortex 11, 37–58 10.1093/cercor/11.1.37 11113034

[B67] Grossberg S. Yazdanbakhsh A. (2005). Laminar cortical dynamics of 3D surface perception: stratification, transparency, and neon color spreading. Vision Res. 45, 1725–1743 10.1016/j.visres.2005.01.006 15792846

[B68] Grossberg S. Yazdanbakhsh A. Cao Y. Swaminathan G. (2008). How does binocular rivalry emerge from cortical mechanisms of 3-D vision? Vision Res. 48, 2232–2250 10.1016/j.visres.2008.06.024 18640145

[B69] Hannagan T. Magnuson J. S. Grainger J. (2013). Spoken word recognition without a TRACE. Front. Psychol. 4:563 10.3389/fpsyg.2013.00563 24058349 PMC3759031

[B70] Healy A. F. (1974). Separating item from order information in short-term memory. J. Verb. Learn. Verb. Behav. 13, 644–655 10.1016/S0022-5371(74)80052-6

[B71] Heeger D. J. (1992). Normalization of cell responses in cat striate cortex. Vis. Neurosci. 9, 181–197 10.1017/S0952523800009640 1504027

[B72] Henson R. N. A. (1996). Unchained memory: error patterns rule out chaining models of immediate serial recall. Q. J. Exp. Psychol. 49, 80–115 10.1080/713755612

[B111] Henson R. N. A. (1998). Short-term memory for serial order: the start-end model. Cognit. Psychol. 36, 73–137 10.1006/cogp.1998.0685 9721198

[B73] Henson R. N. A. (2001). Serial order in short-term memory. The Psychologist 14, 70–73 10632146

[B74] Histed M. H. Miller E. K. (2006). Microstimulation of frontal cortex can reorder a remembered spatial sequence. PLoS Biol. 4:e134 10.1371/journal.pbio.0040134 16620152 PMC1440931

[B75] Hofmann T. Schölkopf B. Smola A. J. (2008). Kernel methods in machine learning. Ann. Stat. 36, 1171–1220 10.1214/009053607000000677

[B76] Houghton G. (1990). The problem of serial order: a neural network model of sequence learning and recall, in Current Research in Natural Language Generation, eds Dale R. Mellish C. Zock M. (London: Academic Press), 287–319

[B77] Hunt R. R. Lamb C. A. (2001). What causes the isolation effect? J. Exp. Psychol. Learn. Mem. Cogn. 27, 1359–1366 10.1037/0278-7393.27.6.1359 11713872

[B78] Knoedler A. J. Hellwig K. A. Neath I. (1999). The shift from recency to primacy with increasing delay. J. Exp. Psychol. Learn. Mem. Cogn 25, 474–487 10.1037/0278-7393.25.2.47415506854

[B114] Lashley K. S. (1951). The problem of serial order in behavior, in Cerebral Mechanisms in Behavior, ed Jeffress L. A. (New York, NY: John Wiley and Sons), 112–136

[B79] McClelland J. L. Elman J. L. (1986). The TRACE model of speech perception. Cogn. Psychol. 18, 1–86 10.1016/0010-0285(86)90015-0 3753912

[B115] McClelland J. L. Rumelhart D. E. (1981). An Interactive Activation Model of context effects in letter perception: Part 1. An account of basic findings. Psychol. Rev. 88, 375–407 10.1037/0033-295X.88.5.375 7058229

[B80] Mhatre H. Gorchetchnikov A. Grossberg S. (2012). Grid cell hexagonal patterns formed by fast self-organized learning within entorhinal cortex. Hippocampus 22, 320–334 10.1002/hipo.20901 21136517

[B81] Miller G. (1956). The magical number seven, plus or minus two: some limits on our capacity for processing information. Psychol. Rev. 63, 81–97 10.1037/h0043158 13310704

[B82] Miller J. L. Liberman A. M. (1979). Some effect of later-occuring information on the perception of stop consonant and semivowel. Percept. Psychophys. 25, 457–465 10.3758/BF03213823 492910

[B83] Murdock B. B. (1962). The serial position effect of free recall. J. Exp. Psychol. 64, 482–488 10.1037/h0045106

[B84] Murdock B. B. Jr. (1968). Serial order effects in short-term memory. J. Exp. Psychol. 76, 1–15 10.1037/h0025694 5654204

[B85] Mushiake H. Saito N. Sakamoto K. Itoyama Y. Tanji J. (2006). Activity in the lateral prefrontal cortex reflects multiple steps of future events in action plans. Neuron 50, 631–641 10.1016/j.neuron.2006.03.045 16701212

[B86] Nieder A. Miller E. K. (2003). Coding of cognitive magnitude: compressed scaling of numerical information in the primate prefrontal cortex. Neuron 37, 149–157 10.1016/S0896-6273(02)01144-3 12526780

[B87] Nieder A. Miller E. K. (2004). A parieto-frontal network for visual numerical information in the monkey. Proc. Natl. Acad. Sci. U.S.A. 101, 74567–77462 10.1073/pnas.0402239101 15123797 PMC409940

[B88] Nipher F. E. (1878). On the distribution of errors in numbers written from memory. Trans. Acad. Sci. 3, 10–11

[B89] Norris D. (1994). Shortlist: a connectionist model of continuous speech recognition. Cognition 52, 189–234 10.1016/0010-0277(94)90043-418426294

[B90] Norris D. McQueen J. M. Cutler A. (2000). Merging information in speech recognition: feedback is never necessary. Behav. Brain Sci. 23, 299–324 10.1017/S0140525X00003241 11301575

[B91] Olson S. Grossberg S. (1998). A neural network model for the development of simple and complex cell receptive fields within cortical maps of orientation and ocular dominance. Neural Netw. 11, 189–208 10.1016/S0893-6080(98)00003-3 12662831

[B92] Page M. Norris D. (1998). The primacy model: a new model of immediate serial recall. Psychol. Rev. 105, 761–781 10.1037/0033-295X.105.4.761-781 9830378

[B93] Pilly P. K. Grossberg S. (2013). How reduction of theta rhythm by medial septum inactivation may covary with disruption of entorhinal grid cell responses due to reduced cholinergic transmission. Front. Neural Circuits 7:173 10.3389/fncir.2013.001173 24198762 PMC3814006

[B94] Postman L. Phillips L. W. (1965). Short-term temporal changes in free recall. Q. J. Exp. Psychol. 17, 132–138 10.1080/17470216508416422

[B95] Raizada R. Grossberg S. (2003). Towards a theory of the laminar architecture of cerebral cortex: computational clues from the visual system. Cereb. Cortex 13, 100–113 10.1093/cercor/13.1.100 12466221

[B96] Repp B. H. (1980). A range–frequency effect on perception of silence in speech. Hask. Lab Stat. Rep. SR–61, 151–165

[B97] Repp B. H. Liberman A. M. Eccardt T. Pesetsky D. (1978). Perceptual integration of acoustic cues for stop, fricative, and affricate manner. J. Exp. Psychol. Hum. Percept. Perform. 4, 621–637 10.1037/0096-1523.4.4.621 722252

[B98] Samuel A. G. van Santen J. P. H. Johnston J. D. (1982). Length effects in word perception: we is better than I but worse than you or them. J. Exp. Psychol. Hum. Percept. Perform. 8, 91–105 10.1037/0096-1523.8.1.91 6460087

[B99] Samuel A. G. van Santen J. P. H. Johnston J. D. (1983). Reply to matthei: we really is worse than you or them, and so are ma and pa. J. Exp. Psychol. Hum. Percept. Perform. 9, 321–322 10.1037/0096-1523.9.2.321 6221077

[B100] Schwab E. C. Sawusch J. R. Nusbaum H. C. (1981). The role of second formant transitions in the stop-semivowel distinction. Percept. Psychophys. 21, 121–128 10.3758/BF03207275 7255090

[B101] Silver M. R. Grossberg S. Bullock D. Histed M. H. Miller E. K. (2011). A neural model of sequential movement planning and control of eye movements: item-order-rank working memory and saccade selection by the supplementary eye fields. Neural Netw. 26, 29–58 10.1016/j.neunet.2011.10.004 22079270

[B102] Tan L. Ward J. A. (2000). A recency-based account of the primacy effect in free recall. J. Exp. Psychol. Learn. Mem. Cogn. 26, 589–625 10.1037/0278-7393.26.6.1589 11185785

[B103] Vitevitch M. S. Luce P. A. (1999). Probabilistic phonotactics and neighborhood activation in spoken word recognition. J. Mem. Lang. 40, 374–408 10.1006/jmla.1998.261810433774

[B104] Von Restorff H. (1933). Über die Wirkung von bereichsbildungen im spurenfeld (the effects of field formation in the trace field). Psychol. Forschung 18, 299–334 10.1007/BF02409636

[B105] Warren R. (1970). Perceptual restoration of missing speech sounds. Science 167, 392 10.1126/science.167.3917.392 5409744

[B106] Warren R. Sherman A. (1974). Phonemic restorations based on subsequent context. Percept. Psychophys. 16, 150–156 10.3758/BF03203268

[B107] Warren R. Warren R. (1970). Auditory illusions and confusions. Sci. Am. 223, 30–36 10.1038/scientificamerican1270-30 5480550

[B108] Wheeler D. C. (1970). Processes in word recognition. Cogn. Psychol. 1, 59–85 10.1016/0010-0285(70)90005-8

[B109] Wickelgren W. A. (1966). Associative intrusions in short-term recall. J. Exp. Psychol. 72, 853 10.1037/h0023884

[B110] Yang S. Heinen S. Missal M. (2008). The effects of microstimulation of the dorsomedial frontal cortex on saccade latency. J. Neurophysiol. 99, 1857–1870 10.1152/jn.00119.2007 18216220

